# Thioredoxin VdTrx1, an unconventional secreted protein, is a virulence factor in *Verticillium dahliae*

**DOI:** 10.3389/fmicb.2023.1130468

**Published:** 2023-03-31

**Authors:** Li Tian, Jing Zhuang, Jun-Jiao Li, He Zhu, Steven J. Klosterman, Xiao-Feng Dai, Jie-Yin Chen, Krishna V. Subbarao, Dan-Dan Zhang

**Affiliations:** ^1^School of Life Science, Qufu Normal University, Qufu, China; ^2^State Key Laboratory for Biology of Plant Diseases and Insect Pests, Institute of Plant Protection, Chinese Academy of Agricultural Sciences, Beijing, China; ^3^National Cotton Industry Technology System Liaohe Comprehensive Experimental Station, The Cotton Research Center of Liaoning Academy of Agricultural Sciences, Liaoning Provincial Institute of Economic Crops, Liaoyang, China; ^4^United States Department of Agriculture, Agricultural Research Service, Salinas, CA, United States; ^5^Western Agricultural Research Center, Chinese Academy of Agricultural Sciences, Changji, China; ^6^Department of Plant Pathology, University of California, Davis, c/o United States Agricultural Research Station, Salinas, CA, United States

**Keywords:** *Verticillium dahliae*, unconventional secreted protein, thioredoxin, ROS scavenging, virulence factor

## Abstract

Understanding how plant pathogenic fungi adapt to their hosts is of critical importance to securing optimal crop productivity. In response to pathogenic attack, plants produce reactive oxygen species (ROS) as part of a multipronged defense response. Pathogens, in turn, have evolved ROS scavenging mechanisms to undermine host defense. Thioredoxins (Trx) are highly conserved oxidoreductase enzymes with a dithiol-disulfide active site, and function as antioxidants to protect cells against free radicals, such as ROS. However, the roles of thioredoxins in *Verticillium dahliae*, an important vascular pathogen, are not clear. Through proteomics analyses, we identified a putative thioredoxin (VdTrx1) lacking a signal peptide. VdTrx1 was present in the exoproteome of *V. dahliae* cultured in the presence of host tissues, a finding that suggested that it plays a role in host-pathogen interactions. We constructed a *VdTrx1* deletion mutant Δ*VdTrx1* that exhibited significantly higher sensitivity to ROS stress, H_2_O_2_, and *tert*-butyl hydroperoxide (*t*-BOOH). *In vivo* assays by live-cell imaging and *in vitro* assays by western blotting revealed that while VdTrx1 lacking the signal peptide can be localized within *V. dahliae* cells, VdTrx1 can also be secreted unconventionally depending on VdVps36, a member of the ESCRT-II protein complex. The Δ*VdTrx1* strain was unable to scavenge host-generated extracellular ROS fully during host invasion. Deletion of *VdTrx1* resulted in higher intracellular ROS levels of *V. dahliae* mycelium, displayed impaired conidial production, and showed significantly reduced virulence on *Gossypium hirsutum*, and model plants, *Arabidopsis thaliana* and *Nicotiana benthamiana*. Thus, we conclude that VdTrx1 acts as a virulence factor in *V. dahliae*.

## 1. Introduction

Reactive oxygen species (ROS), including superoxide anion ($O_{2}^{-}$), hydrogen peroxide (H_2_O_2_), and hydroxyl radical (OH^−^), are generated as natural by-products of oxygen metabolism in aerobes, and also function as part of host repertoire of defense-related activities (Broxton and Culotta, 2016). The critical roles of ROS in defense have been well established in numerous pathosystems (Heller and Tudzynski, 2011; Segal and Wilson, 2018). ROS can react rapidly and nonspecifically with macromolecules, causing molecular damage such as lipid peroxidation, protein oxidation, and mutations (Imlay, 2003). Host ROS accumulation can obstruct pathogen colonization through oxidative cross-linking of cell-walls (Camejo et al., 2016). The oxidative burst, a transient and rapid accumulation of ROS, is part of the initial defense response in higher plants to limit pathogen spread through induction of cell death near the penetration site to cause local necrosis (Lamb and Dixon, 1997).

Since ROS are highly toxic to microbes, pathogens must neutralize or scavenge excess ROS to survive and proliferate within the host. The first line of defense against ROS are the superoxide dismutases (SOD), which catalyze dismutation of $O_{2}^{-}$ to O_2_ and H_2_O_2_, the latter then are removed by catalases, the glutathione system (composed of glutathione peroxidases, glutathione reductases, and glutaredoxins) or the thioredoxin system (composed of peroxiredoxin, thioredoxin, and thioredoxin reductase) (Lu and Holmgren, 2014; Staerck et al., 2017). It comes as no surprise that pathogen survival and virulence has been correlated with the activity of ROS scavenging enzymes in many pathogens. SODs of plant pathogenic fungi, such as *Botrytis cinerea* and *Fusarium graminearum*, play important roles in the detoxification of ROS and pathogenesis (Rolke et al., 2004; Yao et al., 2016). The thioredoxin systems employed by *Magnaporthe oryzae* (Wang et al., 2017) and *Sclerotinia sclerotiorum* to defend against host-generated oxidative stress also contribute significantly to virulence (Rana et al., 2021).

*Verticillium dahliae*, a destructive soilborne phytopathogenic fungus, causes Verticillium wilt disease in a wide range of hosts, including many economically important crops (Klosterman et al., 2009; Chen et al., 2021). As in other pathosystems, the interactions of *V. dahliae* with a variety of hosts are also accompanied by changes in the levels of ROS. For instance, *V. dahliae* triggered H_2_O_2_ production in tomato early in the interaction (Gayoso et al., 2010); ROS production was also detected at infection sites during cotton and *V. dahliae* interactions (Zhang et al., 2017). Furthermore, many *V. dahliae* secreted proteins, such as the elicitor PevD1 (Wang et al., 2012), the PAMPs VdEG1/3 (Gui et al., 2017) and several small cysteine-rich effector proteins (Wang D. et al., 2020) can all induce host ROS accumulation. *V. dahliae* adopts multiple strategies to cope with oxidative stress. Transcription factors Vta2, Som1, and VdHapx relieve ROS stress by regulating expression of genes for oxidative stress response (Tran et al., 2014; Wang Y.L. et al., 2018; Bui et al., 2019). Chromatin remodeling, mediated by a histone-fold protein VdDpb4 and its interacting protein VdIsw2, facilitates the DNA damage repair of *V. dahliae* in response to plant ROS stress (Wang S. et al., 2020). Our previous studies have shown superoxide dismutase family mediating ROS detoxification by scavenging the primary superoxide anion $O_{2}^{-}$ to H_2_O_2_ under conditions of stress (Tian et al., 2021a,b). However, it is unclear whether *V. dahliae* employs other ROS-detoxifying enzyme systems to remove excess H_2_O_2_.

To identify new pathogenicity factors facilitating *V. dahliae* infection, we analyzed the exoproteome of *V. dahliae* induced in cotton tissue-containing medium by iTRAQ (Chen et al., 2016; Wang J. et al., 2018). Using this approach, we identified a predicted thioredoxin (VdTrx1) lacking a signal peptide and containing five highly conserved sites (Chen et al., 2016). The above suggested that VdTrx1 may function in host-pathogen interactions and pathogenicity. Thioredoxins are small, ubiquitous proteins with a redox-active site (WCGPC) conserved throughout evolution (Holmgren, 1989). As hydrogen donors, thioredoxin cycle between their reduced dithiol [Trx-(SH)_2_] and oxidized disulfide (Trx-S_2_) forms to regulate many metabolic enzymes which form disulfide bonds during their catalytic cycle (Vignols et al., 2005). One of them is peroxiredoxin, an enzyme involved in the reduction of H_2_O_2_ (Verdoucq et al., 1999).

Typically, secreted proteins in eukaryotic cells carry an *N*-terminal signal peptide, which direct their sorting to the lumen of the endoplasmic reticulum (ER). They are then transported to the extracellular space through the ER-Golgi route *via* vesicular carriers (Palade, 1975; Ferro-Novick and Brose, 2013). Although lacking an *N*-terminal signal sequence, the secreted human thioredoxin TXN1 was first identified as the adult T-cell leukemia-derived factor (ADF) (Wakasugi et al., 1990). Additionally, thioredoxin can be released from various types of mammalian cells to alter the extracellular redox state (Léveillard and Aït-Ali, 2017). In the plant pathogenic fungus, *Magnaporthe oryzae*, MoTrx2 also plays a critical role in scavenging extracellular ROS during host cell invasion and is likely a secreted protein, although there is no clear evidence for this currently (Wang et al., 2017).

The mechanism of secretion of thioredoxins is unconventional. Secretion of signal peptide-lacking antioxidant superoxide dismutase (SOD1) in *S. cerevisiae* requires a novel membrane compartment called CUPS (compartment for unconventional protein secretion), which marked by the presence of Golgi reassembly and stacking protein (GRASP; Cruz-Garcia et al., 2017). Secretion of *S. cerevisiae* SOD1 is accompanied by the export of thioredoxin such as thioredoxins Trx1/2 and peroxiredoxin Ahp1 in a GRASP-dependent manner (Cruz-Garcia et al., 2020). However, whether CUPS is involved in the secretion of similar antioxidant enzymes lacking a signal peptide in filamentous fungi is not clear.

The main objectives of this study were to: (1) confirm that VdTrx1 is secreted; (2) explore the molecular mechanism of VdTrx1 secretion; (3) determine the role of VdTrx1 in the clearance of intracellular ROS generated by *V. dahliae* itself as well as extracellular host-derived ROS during infection; and (4) determine the role of VdTrx1 in pathogenicity of *V. dahliae*.

## 2. Materials and methods

### 2.1. Fungal culture and plant growth

*V. dahliae* wild-type Vd991 (Chen et al., 2018) originally isolated from cotton was grown on potato dextrose agar solid medium (PDA, potato, 200 g/l; glucose, 20 g/l; agar 15 g/l) or complete medium broth (CM, yeast extract, 6 g/l; casein acids hydrolyzate, 6 g/l; sucrose, 10 g/l) at 25°C. *Agrobacterium tumefaciens* AGL-1 for ATMT transformation of *V. dahliae* was cultured in LB medium at 28°C, as previously described (Wang S. et al., 2016). *Escherichia coli* competent DH5α was used to amplify recombinant plasmids. Cotton (*G. hirsutum* ‘Junmian No. 1’), *N. benthamiana* (LAB), and *Arabidopsis thaliana* (Columbia-0) were grown in a greenhouse of 25°C and 70% relative humidity with a 14 h/10 h, day/night cycle.

### 2.2. Bioinformatics analysis

The DNA and cDNA sequences of *VdTrx1* were retrieved from the genome database of *V. dahliae* (Chen et al., 2018). Homologs of *VdTrx1* in different species were searched by BLASTp using the National Center for Biological Information (NCBI) database.^1^ The gene structure and multiple sequence alignments were determined by the Clustal-W of Bioedit v7.2.0.^2^ Predictions of the domains and functional sites of VdTrx1 were conducted using the CD-Search of NCBI. SignalP5.0 was used to predict signal peptide (Almagro Armenteros et al., 2019). SecretomeP v.2.0 (Bendtsen et al., 2004) and OutCyte 1.0 (Zhao et al., 2019) were used for predictions of unconventional secretion of VdTrx1.

### 2.3. Fungal transformation

To construct the *VdTrx1* knockout mutant, the upstream and downstream fragments of *VdTrx1* were amplified from the Vd991 genome, respectively. The hygromycin phosphotransferase gene cassette, which was amplified from the pUC-*Hyg*, the upstream and downstream fragments of *VdTrx1* were ligated together by fusion PCR and integrated into the *EcoR*I*/Hind*III sites of binary vector pGKO2 (Khang et al., 2005). Positive gene deletion strains were selected on PDA with 200 μg/ml cefotaxime, 50 μg/ml hygromycin, and 200 μg/ml 5-fluoro-2′-deoxyuridine. To obtain the complemented strain, a fragment containing 1 kb 5′-upstream of the *VdTrx1* coding region, the *VdTrx1* coding sequence, and 0.5 kb of the *VdTrx1* 3′-flanking region was amplified from the genomic DNA of Vd991 and ligated into the pCOM vector (Zhou et al., 2013). Complemented strains were selected on PDA with 200 μg/ml cefotaxime and 50 μg/ ml Geneticin (G418). To create *VdTrx1* overexpression strains, the CDS sequence of *VdTrx1* was cloned from the cDNA of Vd991. The fragment was integrated into the *Kpn*I site of the pCOM-GFP vector to construct the VdTrx1-GFP fusion protein (Tian et al., 2021b). The CDS region of VdTrx1 linked with the HA tag was integrated into the *Sac*I*/Xba*I sites of the pCOM-TrpC vector to construct the VdTrx1-HA fusion protein (Zhou et al., 2013). The recombinant plasmids were transformed into the wild-type strain Vd991, and the mutant strains Δ*VdGRASP,* Δ*VdATG1*, and Δ*VdVps36*. Primers used for expression profiling are listed in Supplementary Table S1.

### 2.4. Vegetative growth and conidiation assays

To observe the vegetative growth phenotype of fungi, 2 μl of the conidial suspension with a concentration of 5 × 10^6^ conidia/ml were cultured on PDA, CM and MM (6 g/l NaNO_3_, 0.52 g/l KCl, 0.52 g/l, MgSO_4_.7H_2_O, 1.52 g/l KH_2_PO_4_, 10 g/l glucose, 0.001% (w/v) thiamine and 0.1% (w/v) trace elements) medium at 25°C for 12 days. For sulfur utilization analysis, inorganic sulfur sulfite (${\mathrm{SO}}_{3}^{2-}$) and organic sulfur L(+)-Cysteine (Coolaber) were added to MM medium at concentrations of 2 mM and 1.4 mM, respectively and growth was analyzed.

To analyze the sporulation of *V. dahliae*, the fungus was grown on PDA plates for 9 days. Three 5-mm-diameter blocks of the fungus were cut from the edge of the colony, placed in 1 ml of sterile water, and turbine oscillated for 1 min. The numbers of conidia were counted using a hemocytometer.

### 2.5. Stress and penetration analysis

The different concentrations of H_2_O_2_ (1.0 mM, 1.5 mM, and 2.0 mM) and *tert*-Butyl hydroperoxide (*t*-BOOH) (0.2 mM, 0.3 mM and 0.4 mM) (Sigma-Aldrich) in the CM solid medium were used to detect the sensitivity of WT, *VdTrx1* mutant, and *VdTrx1* complemented strains to oxidative stress. Otherwise, 1 M sorbitol (Solarbio) and 200 μg/ml Congo red (Sigma-Aldrich) were added to the CM medium to assay osmotic stress and cell-wall integrity.

To measure the penetration ability of the wild type, the *VdTrx1* mutant, and the *VdTrx1* complemented strains, 5 μl of the spore suspensions of each, with concentrations of 5 × 10^6^ conidia/ml, was cultured on MM plate covered with sterilized cellophane membrane and incubated at 25°C. The cellophane membranes were removed after 4 days. The plates were further cultured for 3 days at 25°C to observe the hyphae that had passed through the membrane forming colonies.

### 2.6. Yeast signal sequence trap system

Functional validation of the *N*-terminal peptide of VdTrx1 was performed as previously described (Jacobs et al., 1997). The nucleotide sequence of *VdTrx1* encoding the *N*-terminal 25 amino acids and CDS regions was cloned into the pSUC2 vector, and the resulting plasmids, pSUC2-VdTrx1^N25^ and pSUC2-VdTrx1, were transformed into the yeast strain YTK12. Transformants were screened on CMD-W (lacking tryptophan) medium for 2 days at 30°C. The positive transformants, the negative controls (YTK12 and YTK12::pSUC2), and the positive control (pSUC2-SP^Avr1b^) were incubated with 10 mM acetic acid–sodium acetate solution (pH = 4.7) and 10% sucrose medium at 37°C for 10 min. The supernatant was collected and incubated with 0.1% 2,3,5-triphenyl tetrazolium chloride (TTC) for 10 min. Invertase enzymatic activity was confirmed by an increase in insoluble red-colored triphenyl formazan due to the reduction of TTC.

### 2.7. Protein extraction and western blot

The mycelium of *V. dahliae* (WT::VdTrx1-HA, Δ*VdGRASP*:: VdTrx1-HA, Δ*VdVps36*::VdTrx1-HA, and Δ*VdATG1*::VdTrx1-HA) in CM medium was ground into powder and suspended in the extraction buffer (RIPA lysis buffer: 500 μl; phenylmethylsulfonyl fluoride (PMSF), 1 mM). Centrifugation at 12,000 rpm for 10 min at 4°C yielded total protein in the supernatant. For the extraction of secreted proteins, strains were cultured in CM medium for 5 days and transferred to MM medium for 3 days to collect the supernatant. The secreted proteins were fractionated from the supernatant by phenol extraction and stored in 80% acetone (Wang et al., 2011). The samples were separated using 12% SDS-PAGE and then transferred to Immobilon-P transfer membranes (Merck Millipore). The membranes were incubated with anti-HA (Abmart, M20003, 1:5000) and anti-β-actin (Abclonal, AC006, 1:2000), then goat anti-mouse IgG-HRP (TransGen Biotech, HS201-01, 1:5000) and goat anti-rabbit IgG-HRP (TransGen Biotech, HS101-01, 1:5000) as secondary antibody, respectively. Chemiluminescence was detected with Immobilon Western Chemiluminescent HRP Substrate (Merck Millipore).

### 2.8. Confocal fluorescence microscopy

The onion intraepidermal cells were soaked in the spore suspensions of the *V. dahliae* strains WT::VdTrx1-GFP, Δ*VdGRASP*::VdTrx1-GFP, Δ*VdATG1*::VdTrx1-GFP, and Δ*VdVps36*:: VdTrx1-GFP for 30 min, and the onion intraepidermal cells were placed on 1% (w/v) water agar for 5 days at 25°C. The WT::VdEG1-GFP strain-infected onion epidermal cells were used as the positive control, while the WT::GFP strain-infected onion epidermal cells were used as the negative control. Fluorescence of GFP fusion protein was observed by laser confocal microscope (ZEISS, LSM 880) at emission and reception wavelengths of 488 and 510 nm.

### 2.9. Reactive oxygen species staining

The 3,3′- diaminobenzidine (DAB) was used as the stain to detect the reactive oxygen species (ROS) in cotton during the infection of *V. dahliae.* Briefly, sterile cotton seeds were placed on wet filter paper, and the germinated cotton sprouts were soaked in the sterile water (CK) or the conidial suspensions of Vd991, Δ*VdTrx1*, and EC strains at a concentration of 1 × 10^7^ conidia/ml for 30 min after 4 days. The radicles soaked in water or conidial suspensions were then incubated on wet filter paper for 2 days and 5 days, respectively. The radicles were placed in the DAB staining solution (0.1% wt/vol DAB; Aladdin) in the dark and decolored with 95% ethanol for 20 min. Samples were subsequently examined under light microscopy. For the detection of intracellular ROS levels, the strains were cultured in CM medium for 4 days and stained with 50 μM 2′,7′ –dichlorofluorescein diacetate (DCFH-DA, Sigma) for 20 min. The mycelium was rinsed three times with 1 × phosphate-buffered saline (PBS) and observed by confocal microscopy (Leica, TCS SP8).

### 2.10. RT-qPCR

To analyze the transcription levels of *VdTrx1* in response to H_2_O_2_, the Vd991 strain was cultured in CM liquid medium for 4 days, and switched to incubate in CM liquid medium containing 1 mM hydrogen peroxide (H_2_O_2_) for 3 h. Total RNA was isolated from fungal mycelium by using a total RNA Miniprep kit (Aidlab). The cDNA was reverse transcribed using TransScript II One-Step gDNA Removal and cDNA Synthesis SuperMix (TransGen Biotech). The transcription levels of *VdTrx1* relative to the constitutively expressed elongation factor 1-α of *V. dahliae* were quantified using the 2^−∆∆Ct^ method (Livak and Schmittgen, 2001).

To analyze the temporal pattern of *VdTrx1* expression, 1 × 10^8^ conidia/ml of wild-type strain was used to inoculate cotton. The roots of cotton were collected at 0, 0.5, 1, 2, 3, 4, 5, and 6 dpi, respectively. Total RNA of roots was extracted, and the qVdTrx1-F/R were used as the primers to detect the expression of *VdTrx1* during infection by using the 2^−∆∆Ct^ method (Livak and Schmittgen, 2001). The amplification reaction was carried out using 2 × Taq Pro Universal SYBR qPCR Master Mix (Vazyme). The experiment method included pre-denaturation at 95°C for 10 min, followed by 40 cycles of 95°C denaturation for 15 s, 60°C annealing for 30 s, and 72°C extension for 30 s. Primers used for expression profiling are listed in Supplementary Table S1.

### 2.11. Pathogenicity assays

Spore suspensions of the wild-type, mutant, and complemented strains at a concentration of 1 × 10^7^ conidia/ml each, were prepared for pathogenicity tests. Three-week-old susceptible cotton seedlings (*G. hirsutum* ‘Junmian No. 1’), *N. benthamiana* and *Arabidopsis thaliana* (Col-0) were inoculated as previously described methods (Zhang et al., 2019; Sun et al., 2021). Twenty cotton plants, five *N. benthamiana* plants and five *Arabidopsis thaliana* plants were inoculated with each strain. The symptoms of Verticillium wilt were photographed 3 weeks later. Then cotton stems were cut longitudinally to observe vascular bundle browning. The vascular bundle browning in stems of at least ten cotton plants was observed.

For analyses of fungal biomass by qPCR, root-stem junctions of cotton and *N. benthamiana*, and the whole *Arabidopsis thaliana* plants were collected for DNA extraction. The quantification of *V. dahliae* DNA was carried out using the *V. dahliae* elongation factor 1α (*VdEF- 1α*) with the cotton 18S rRNA (*Gh18S*), *N. benthamiana EF- 1α* (*NbEF-1α*), or *A. thaliana* ubiquitin-specific protease 1 (*AtUBQ1*) genes used in normalization using the 2^−∆∆Ct^ method. The amplification reaction was carried out using 2 × Taq Pro Universal SYBR qPCR Master Mix (Vazyme). The experiment method included pre-denaturation at 95°C for 10 min, followed by 40 cycles of 95°C denaturation for 15 s, 60°C annealing for 30 s, and 72°C extension for 30 s. The primers used are listed in Supplementary Table S1.

## 3. Results

### 3.1. *VdTrx1* encodes a thioredoxin lacking a signal peptide

**Figure 1:**
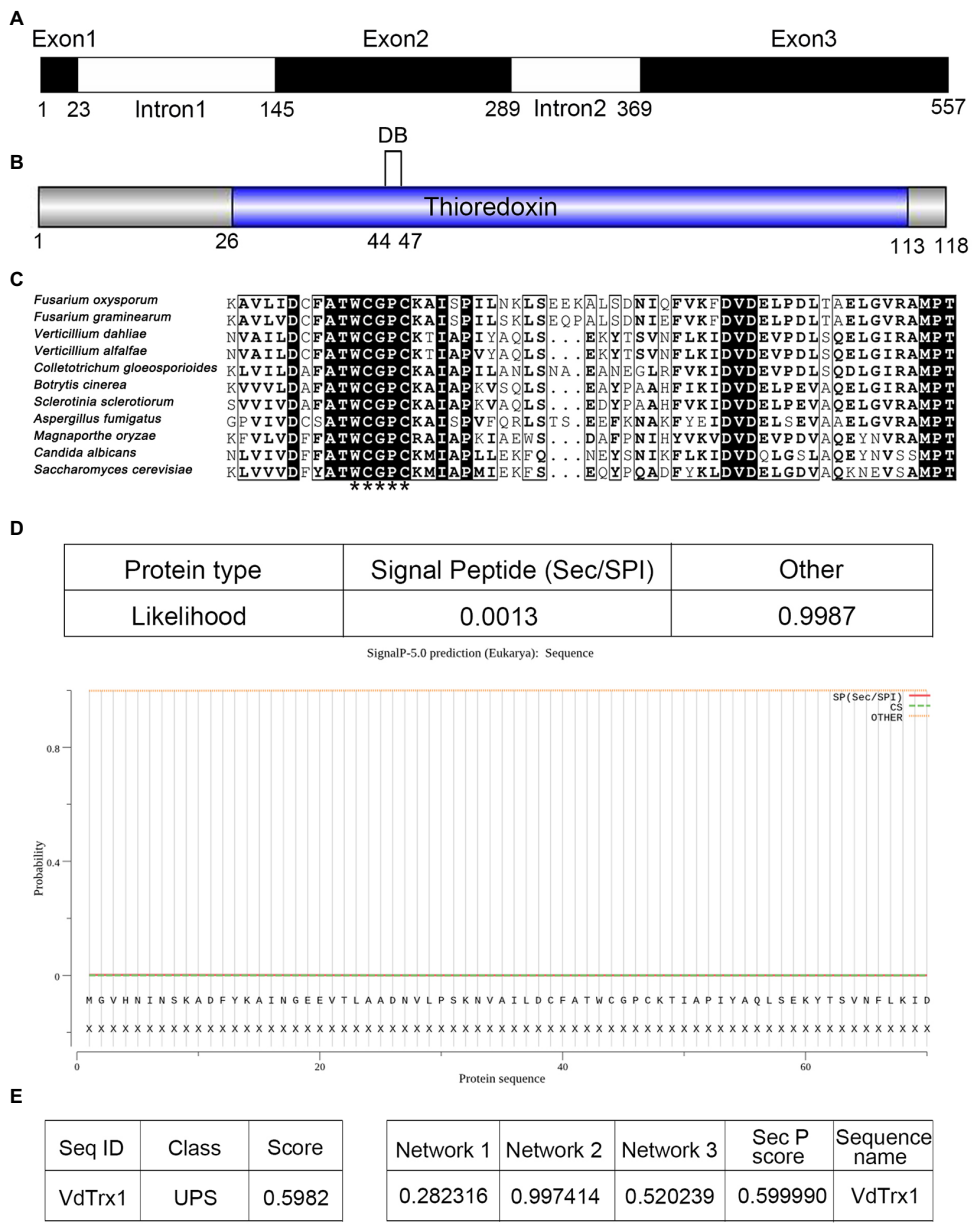
Gene cloning and bioinformatic analysis of VdTrx1 from *Verticillium dahliae*. **(A)** Structure of *VdTrx1*. Exons are black boxes and introns are white boxes. **(B)** Conserved domain of VdTrx1 predicted by CD-Search of NCBI. DB represent disulfide bond. **(C)** Alignment of conserved region of thioredoxins. Thioredoxin-specific redox-active sites are marked by asterisks. GenBank accession number of aligned sequences are *V. dahliae* (VEDA_00080); *Verticillium alfalfae* (XP_003005517.1); *Fusarium oxysporum* (EGU81922.1); *Fusarium graminearum* (ESU16261.1); *Magnaporthe oryzae* (EHA47211.1); *Botrytis cinerea* (EMR86794.1); *Aspergillus fumigatus* (XP_753517.1); *Colletotrichum gloeosporioides* (EQB49288.1); *Candida albicans* (XP_719372.1); *Saccharomyces cerevisiae* (YLR043C). **(D)** Signal peptide prediction of VdTrx1 using SignalP 5.0 program. OutCyte 1.0 **(E)** and SecretomeP 2.0 **(F)** were used to predict unconventional secretion characteristic of VdTrx1. UPS, unconventional protein secretions; Network 1, score of amino acid composition; Network 2, score of secondary structure prediction; Network 3, score of transmembrane helix prediction; SepP score, the average of three network scores.

Exoproteome analysis of *V. dahliae* Vd991 induced by a cotton-containing medium revealed a putative thioredoxin VdTrx1 (VEDA_00080, Gene-ID in VdLs.17 genome: VDAG_09916) in the extracellular proteins of *V. dahliae* (Chen et al., 2016). The 557 bp full-length *VdTrx1* gene contains three exons of 23, 145 and 189 bp and two introns of 121 and 79 bp (Figure 1A). The *VdTrx1* ORF encodes a polypeptide of 118 amino acids with a predicted molecular weight of 12.89 kDa and an isoelectric point of 4.77. Searches for conserved domains within VdTrx1 by CD-Search of NCBI identified a typical thioredoxin motif (pfam00085, from N-26 to L-113) (Figure 1B). The amino acid alignment of the partial conserved domain of VdTrx1 with other phytopathogen Trx1 orthologs showed they all contain the thioredoxin-specific redox-active site WCGPC (Figure 1C). SignalP analysis showed with a high degree of probability (0.9987, Figure 1D) that the protein sequence of VdTrx1 lacks an *N*-terminal signal peptide. However, VdTrx1 was predicted to be a potential non-conventional secreted protein, as predicted by OutCyte 1.0 (with UPS score of 0.598, above the threshold of 0.5) (Figure 1E) and SecretomeP 2.0 (with SecP scores of 0.599, above the threshold of 0.5) (Figure 1F). Together, these results suggested that VdTrx1 is likely a thioredoxin lacking a signal peptide, which is exported into the extracellular space unconventionally.

### 3.2. VdTrx1 has thioredoxin activity and contributes to the oxidative stress tolerance

**Figure 2:**
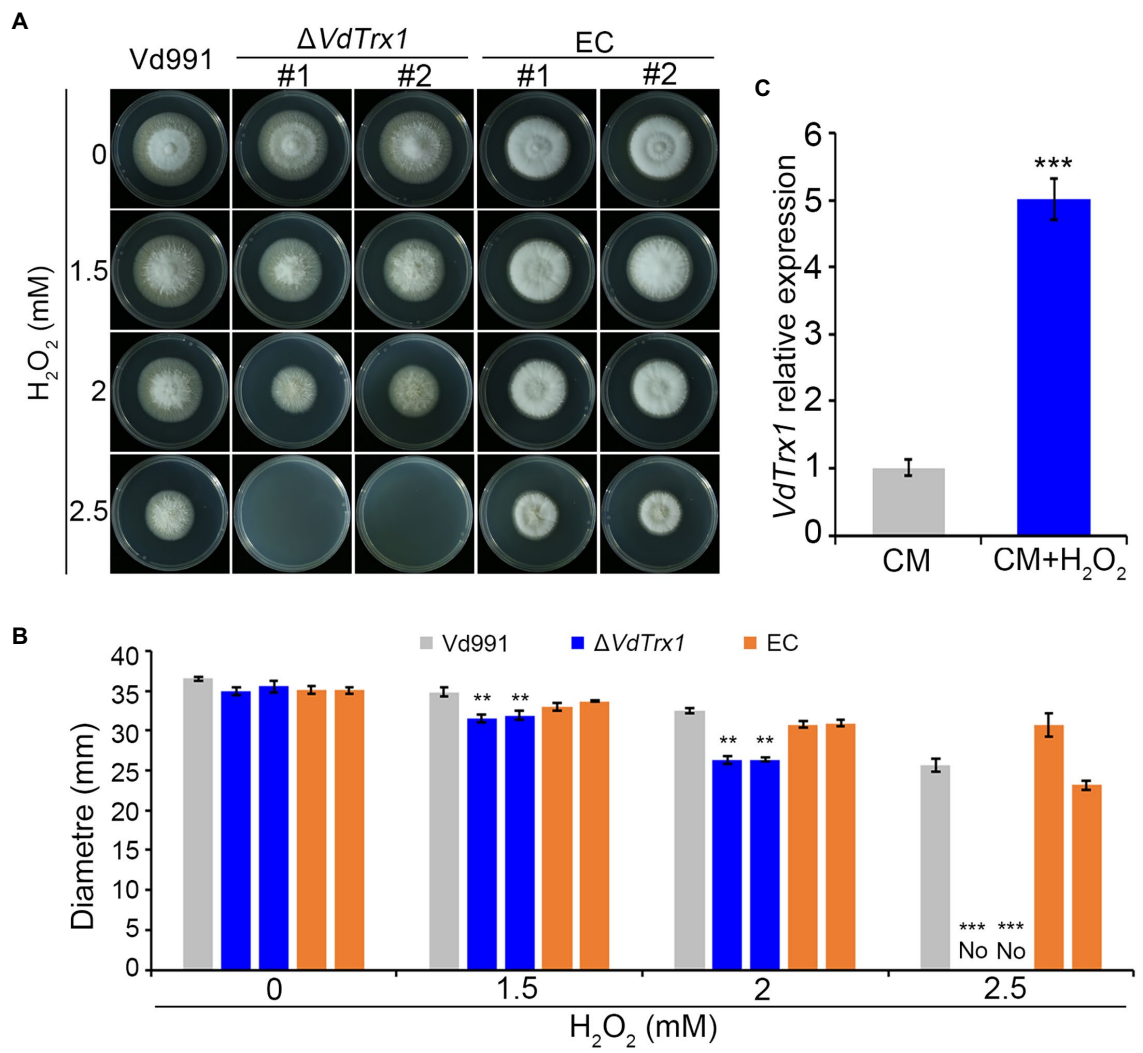
VdTrx1 of *V. dahliae* mediates the response to oxidative stress. **(A)** Radial growth of *VdTrx1* deletion strains, complemented strains (EC#1 and #2) and wild-type strain (Vd991) on CM (complete medium) supplemented with H_2_O_2_ at specified concentrations for 9 days. **(B)** Colony diameters of various *V. dahliae* strains on CM plates containing different concentration of H_2_O_2_ following 9 days incubation. Means and standard deviations of the mean from three biological replicates are shown. Asterisks (**) and (***) denote significant differences *p* < 0.01 and *p* < 0.001, respectively, according to Student’s *t*-test. **(C)** Reverse transcription-quantitative PCR (RT-qPCR) of *VdTrx1* transcripts in *V. dahliae* hyphae treated with 1.0 mM H_2_O_2_ or incubated in CM only for 3 h. Total cDNA abundance in the samples was normalized to using *VdEF-1α* gene as a control. In RT-qPCR, expression of *VdTrx1* in the strain without H_2_O_2_ treatment was set as 1. Bars indicate standard deviations from three biological replicates, *** denotes significant differences at *p* < 0.001 (Student’s *t*-test).

To study the biological function of *VdTrx1*, gene deletion mutants were constructed by replacing the coding sequence of *VdTrx1* in the wild-type strain Vd991 genome with a hygromycin resistance cassette *via* homologous recombination. Deletion of the *VdTrx1* ORF was confirmed by PCR in five candidate transformants and two of them were arbitrarily selected for further characterization (Supplementary Figure S1A). Complemented strains (EC-1/2) were generated by reintroducing *VdTrx1* with its native promoter and a neomycin resistance cassette into Δ*VdTrx1*-1/2 (Supplementary Figure S1B), respectively. The radial growth of the Δ*VdTrx1* strains on complete medium (CM) was similar to that of the wild-type strain (Figure 2A).

Considering that a well-established cellular function of the thioredoxin is to neutralize ROS and therefore contribute to oxidative stress resistance (Meyer et al., 2009), the hyphal growth under H_2_O_2_ stress between the wild-type and Δ*VdTrx1* strains was then compared. As shown in Figures 1A,B, growth of *∆VdTrx1* on CM supplemented with 1.5 and 2 mM H_2_O_2_ was significantly inhibited, with colony diameters reduced by 10 and 26%, respectively, relative to those of the wild-type strain. Hyphal growth was halted at 2.5 mM H_2_O_2_, for the *∆VdTrx1* strain but not the wild-type strain (Figures 2A,B). Reintroduction of *VdTrx1* into the Δ*VdTrx1* strain restored the H_2_O_2_ resistance to the wild-type level (Figures 2A,B).

The relative expression level of *VdTrx1* under H_2_O_2_ stress was assessed *via* reverse transcription quantitative PCR (RT-qPCR). After treatment for 3 h with 1 mM H_2_O_2_, expression of *VdTrx1* was significantly upregulated 5-fold in the wild-type strain (Figure 2C). Moreover, upon exposure to another ROS stress, *t*-BOOH, the Δ*VdTrx1* strain also displayed increased sensitivity, but this sensitivity was reduced by complementation (Supplementary Figures S2A,B). Together, these results indicate that VdTrx1 has thioredoxin activity and contributes to oxidative stress tolerance in *V. dahliae*.

### 3.3. VdTrx1 can be secreted by *Verticillium dahliae*

**Figure 3:**
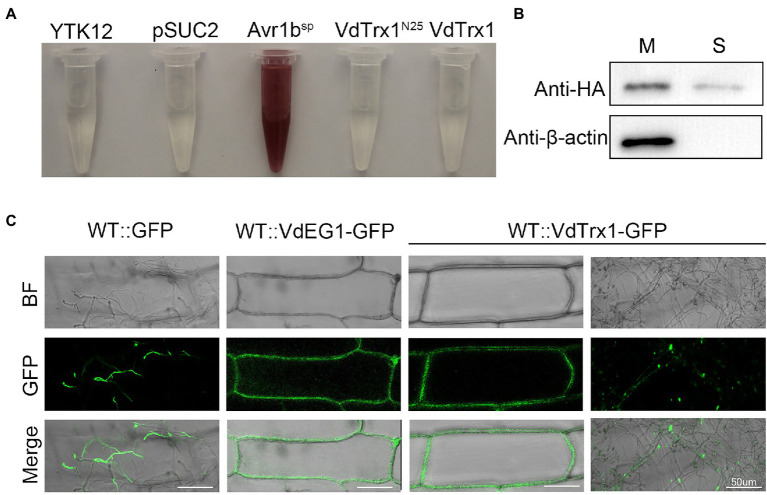
VdTrx1 can be secreted by *V. dahliae* to extracellular spaces. **(A)** Validation of the non-secretory function of the 25 aa *N*-terminal peptide and full-length sequence of VdTrx1 by a yeast signal trap assay. The region encoding the 25 aa *N*-terminal peptide and the full-length sequence of *VdTrx1* were fused in-frame to the invertase sequence in the pSUC2 vector and transformed into yeast strain YTK12. The untransformed YTK12 strain and empty pSUC2 vector transformed into YTK12 were used as negative controls, while the pSUC2-Avr1b^SP^ vector (integrating known functional signal peptide Avr1b^SP^ into pSUC2) transformed YTK12 was used as a positive control. Only yeast strains that can secrete invertase converted 2,3,5- triphenyl tetrazolium chloride (TTC) to red triphenylformazan. **(B)** Western blotting assay demonstrates VdTrx1 secretion into culture filtrates. VdTrx1-HA was expressed in *V. dahliae* wild-type strain Vd991 to produce strain WT::VdTrx1-HA. Proteins extracted from mycelia (M) and culture supernatants (S) of strain WT::VdTrx1-HA were analyzed using western blotting with anti-HA or anti-β-actin antibodies, as indicated. **(C)** Live-cell imaging by confocal microscopy of VdTrx1. *V. dahliae* strains WT::VdTrx1-GFP, WT::GFP and WT::VdEG1-GFP were used to infect onion epidermal cells (the latter two strains were used as controls), respectively. Images were taken at 5 dpi using confocal laser scanning microscopy to perform a subcellular localization assay. Bar, 50 μm.

To verify the *N*-terminal of VdTrx1 does not contain the functional signal peptide, as predicted by SignalP 5.0, we examined VdTrx1 using a yeast signal sequence trap assay. The region encoding 25 amino acids of the *N*-terminus of VdTrx1 was fused to the vector pSUC2, which carries an invertase gene lacking the region encoding a signal peptide, to generate pSUC2-VdTrx^N25^. Invertase secretion was tested with a 2,3,5-triphenyl tetrazolium chloride (TTC) assay, in which secreted invertase catalyzes TTC in medium into the insoluble red compound. As expected, pSUC2-VdTrx1^N25^ did not result in the catalysis of TTC in the medium (Figure 3A), indicating that the 25 aa *N*-terminal peptide of VdTrx1 is not a functional signal peptide.

Since the bioinformatic analyses predicted VdTrx1 to be an unconventionally secreted protein (Figure 1), we tested experimentally whether VdTrx1 can be exported to the extracellular space. For this purpose, we first generated WT::VdTrx1-HA, a strain expressing a *C*-terminally HA-tagged VdTrx1 in the wild-type background (Supplementary Figure S3). To examine whether VdTrx1 is secreted, we performed western blotting of the culture supernatants of this VdTrx1-HA expressing strain using anti-HA antibody. Meanwhile, to eliminate the possibility that the VdTrx1-HA was detected in the culture supernatants due to cell lysis, intracellular cytoskeletal protein β-actin of *V*. *dahliae* was used as a control. As shown in Figure 3B, VdTrx1-HA was detected in both the mycelia and the culture supernatants of WT::VdTrx1-HA. In contrast, the β-actin protein was only clearly detected in mycelial protein extracts but not in culture supernatants under the same experimental conditions. These results therefore suggest that VdTrx1 likely is secreted into culture medium *in vitro*.

To further confirm if VdTrx1 is secreted *in vivo*, we examined the subcellular location of VdTrx1 using an onion epidermal cell system. When the onion epidermal cells were inoculated with the WT::GFP strain expressing free GFP, green fluorescence was detected in the fungal hyphae or conidia, but not in the onion epidermal cells since GFP alone cannot be secreted into the extracellular space (Figure 3C). Signal-peptide containing VdEG1 is a known apoplastic elicitor secreted by *V. dahliae* during infection (Gui et al., 2017). In this experiment, VdEG1-GFP was observed around the plasma membrane in onion epidermal cells (Figure 3C). After incubation with the WT::VdTrx1-GFP strain expressing VdTrx1-GFP, the fluorescence was clearly detected both in the hyphal and the extracellular space around the onion epidermal cell plasma membrane (Figure 3C). Together, these results indicate that VdTrx1 is not only an intracellular protein of *V. dahliae*, but also can be secreted extracellularly and translocated into the host apoplastic space.

### 3.4. Putative secretion mechanism of VdTrx1

**Figure 4:**
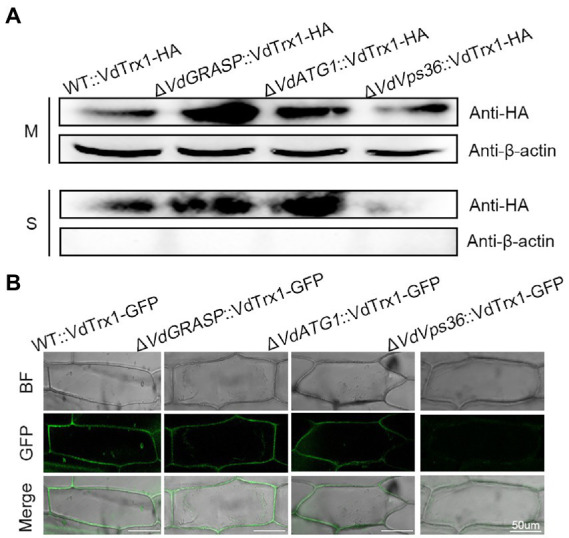
Secretion mechanism of VdTrx1 from *V. dahliae*. **(A)** Proteins extracted from mycelia (M) and culture supernatants (S) of strain WT::VdTrx1-HA, Δ*VdGRASP*::VdTrx1-HA, Δ*VdATG1*::VdTrx1-HA and Δ*VdVps36*::VdTrx1-HA were analyzed using western blotting with anti-HA or anti-β-actin antibodies, as indicated. **(B)** The *V. dahliae* strains WT::VdTrx1-GFP, Δ*VdGRASP*::VdTrx1-HA, Δ*VdATG1*::VdTrx1-HA and Δ*VdVps36*::VdTrx1-HA were used to inoculate onion epidermal cells, respectively, at room temperature for 5 days followed by confocal microscopy imaging. Bar, 50 μm.

We further investigated the molecular mechanism of VdTrx1 secretion. In *S. cerevisiae*, secretion of superoxide dismutase (SOD1) lacking a signal peptide requires a novel membrane compartment called CUPS (compartment for unconventional protein secretion), which contains Golgi reassembly and stacking protein (GRASP), autophagy-related proteins, and proteins of the endosomal sorting complex required for transport (ESCRT) machinery (Cruz-Garcia et al., 2017). Further, secretion of the *S. cerevisiae* thioredoxin (Trx1) lacking a signal-peptide also requires GRASP (Cruz-Garcia et al., 2020). Thus, we tested the involvement of VdGRASP in the secretion of VdTrx1. For this purpose, we deleted the *VdGRASP* gene in *V. dahliae* (*VdGRASP*, VEDA_09322, Gene-ID in VdLs.17 genome: VDAG_03042) and introduced the construct VdTrx1-HA into the Δ*VdGRASP* strain, resulting in a strain overexpressing VdTrx1-HA in the *VdGRASP* deletion mutant. However, as in the wild-type strain, we observed that VdTrx1 was also present in the supernatants derived from the Δ*VdGRASP*::VdTrx1-HA strain (Figure 4A). Moreover, the onion cells inoculated with Δ*VdGRASP*::VdTrx1-GFP, a strain expressing VdTrx1-GFP in Δ*VdGRASP* background, also showed aggregation of fluorescence at the periphery of the onion epidermal cells, similar to that of WT::VdTrx1-GFP (Figure 4B). These results indicate that export of VdTrx1 is independent of VdGRASP.

We examined whether unconventional secretion of VdTrx1 is dependent on autophagy or ESCRT machinery. With a similar strategy, we deleted the *VdATG1* (*VdATG1*, VEDA_06789, Gene-ID in VdLs.17 genome: VDAG_05745), which functions at the initial stage of autophagy, and *VdVps36* (*VdVps36*, VEDA_06789, Gene-ID in VdLs.17 genome: VDAG_05745), the coding product of which is a member of ESCRT-II complex in *V. dahliae*. After expressing VdTrx1-HA in the *VdATG1* and *VdVps36* deletion background, we observed that the VdTrx1-HA signal was absent in the supernatant of the *VdVps36* mutant, but not in the supernatant of the *VdATG1* mutant (Figure 4A). These results are consistent with our microscopic observations that after inoculating Δ*VdATG1*::VdTrx1-GFP (a strain expressing VdTrx1-GFP in *VdATG1* deletion background) and Δ*VdVps36*::VdTrx1-GFP (a strain expressing VdTrx1-GFP in *VdVps36* deletion background) on onion cells, GFP fluorescence around the onion epidermal cells disappeared in background of *VdVps36* mutant rather than *VdATG1* mutant (Figure 4B). Taking together, we conclude that the extracellular release of VdTrx1 is dependent on VdVps36 but not VdGRASP and VdATG1, and the secretion pathway of VdTrx1 in *V. dahliae* does not require the same set of components as Trx1 in *S. cerevisiae*. Moreover, the full-length coding sequence of VdTrx1 fused in-frame to invertase was not secreted from yeast, (Figure 3A), further confirming that the unconventional VdTrx1 secretion pathway in *V. dahliae* is not the same as in *S. cerevisiae*.

### 3.5. VdTrx1 functions in neutralizing endogenous and exogenous ROS

**Figure 5:**
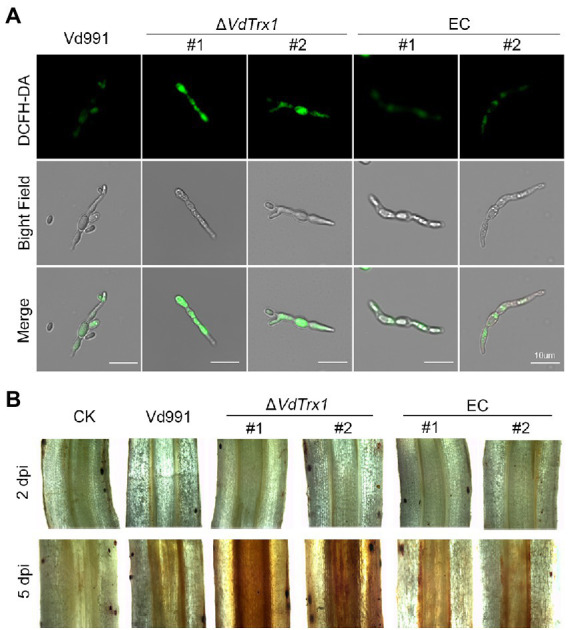
The role of VdTrx1 from *V. dahliae* in the degradation of ROS. **(A)** Hyphae from wild-type strain Vd991, *VdTrx1* deletion strains, and the complemented strains were grown in liquid complete medium for 4 days. The fluorescent dye 2′,7′–dichlorofluorescin diacetate (DCFH-DA) was used to visualize the production of H_2_O_2_. Bar, 10 μm. **(B)** Accumulation of H_2_O_2_ in infected cotton roots. Detection of H_2_O_2_ accumulation in cotton roots inoculated with sterile water, the wild-type strain Vd991, Δ*VdTrx1* and complemented strains at 2 days and 5 days post-inoculation, respectively, by 3,3′- diaminobenzidine staining.

Since VdTrx1 has thioredoxin activity and is distributed both intracellularly and extracellularly, we investigated its role in scavenging endogenous and exogenous ROS. Intracellular levels of ROS were determined by DCFH-DA staining of *V. dahliae* mycelium after culturing in CM medium for 4 days. As shown in Figure 5A, the mycelia of the *VdTrx1* deletion mutant displayed stronger bright green fluorescence compared to the mycelia of wild-type and complemented strains, indicating that the loss of VdTrx1 accompanied by increased accumulation of endogenous ROS and VdTrx1 functions in degrading intracellular ROS.

We further examined whether VdTrx1 could neutralize extracellular ROS generated by the host during infection. For this purpose, the change in ROS in cotton roots infected by *V. dahliae* strains was detected by DAB (3,3′-diaminobenzidine) staining, which produces a reddish-brown stain when oxidized by H_2_O_2_. In the relative early stages of infection (2 dpi), cotton roots inoculated by all strains displayed similar phenotypes, showing very few reddish-brown spots following DAB staining (Figure 5B). Potentially, few *V. dahliae* hyphae entered the root tissue at 2 dpi. In contrast, after 5 dpi, when the invading hyphae entered the xylem vessels and induced host-derived ROS generation (Tian et al., 2021b; Tian and Kong, 2022), cotton roots inoculated with the Δ*VdTrx1* strain showed increased accumulation of H_2_O_2_ in comparison with that of the wild-type strain, but decreased accumulation of H_2_O_2_ was restored in the complemented strains (Figure 5B). Together, these results indicated that VdTrx1 functions in apoplast to neutralize host-derived ROS and plays roles in scavenging both endogenous and exogenous ROS produced in mycelia and in host defense, respectively.

### 3.6. VdTrx1 is essential for sulfite assimilation and conidiation in *Verticillium dahliae*

**Figure 6:**
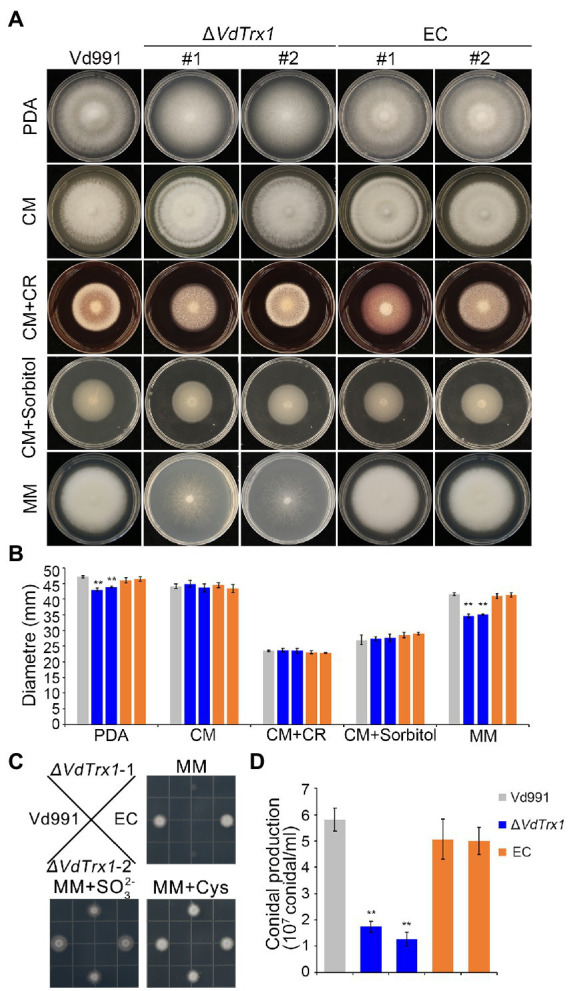
VdTrx1 is essential for sulfite assimilation and conidiation in *V. dahliae*. **(A)** Radial growth of *VdTrx1* deletion strains, complemented strains (EC#1 and #2) and wild-type strain (Vd991) on different media after incubation at 25°C for 12 days. A 2 μl conidial suspension (5 × 10^6^ conidia/ml) was placed in the center of the plates as inoculum. **(B)** Colony diameter of the different strains on different media after 12 days of incubation. Means and standard deviations from three biological replicates are shown. Double asterisks indicate significant differences at *p* < 0.01. **(C)** Growth of *V. dahliae* strains on plates with different source of sulfate. The various *V. dahliae* strains were inoculated on MM plates, or MM plates supplemented with 2 mM sulfate (${\mathrm{SO}}_{3}^{2-}$) and 1.4 mM cysteine (distribution of strains on the plate are indicated in figure). The strains were cultured at 25°C for 4 days. **(D)** Quantification of conidial production was based on a 5-mm-diameter PDA agar plug from the edge of 9-days-old fungal culture colonies in 1 ml water. The error bars represent the standard deviations of the mean (n = 3). Student’s *t*-test was employed to determine treatment differences and double asterisks indicate significant differences at *p* < 0.01.

The *VdTrx1* deletion mutants showed slightly reduced radial growth on potato dextrose agar (PDA) medium when compared with the wild-type strain and complemented transformants. However, the Δ*VdTrx1* strain displayed no obvious mycelial growth defect when cultured in nutrient-rich CM medium (Figures 6A,B). To investigate the function of VdTrx1 in the response to cell-wall and hyperosmotic stress, the relevant *V. dahliae* strains were grown on CM supplemented with 200 μg/ml Congo red and 1 M sorbitol, respectively. As shown in Figures 6A,B, sensitivity of the Δ*VdTrx1* strain to the cell-wall and hyperosmotic stresses was comparable to that observed in wild-type strain Vd991, suggesting VdTrx1 is not essential for the response to cell-wall and hyperosmotic stress in *V. dahliae*.

Thioredoxin is an efficient reductant for 3′-phosphoadenosine 5′-phosphosulfate (PAPS) reductase, the enzyme involved in the reduction process of ${\mathrm{SO}}_{4}^{2-}$ to ${\mathrm{SO}}_{3}^{2-}$ (Berendt et al., 1995), which is a key step in sulfate assimilation. To investigate whether VdTrx1 participates in the sulfate assimilation process, we tested the growth rate of Δ*VdTrx1* in MM medium, which only contains ${\mathrm{SO}}_{4}^{2-}$. Radial growth of Δ*VdTrx1* was reduced by 20% relative to the wild-type strains and aerial hyphae production was severely impaired due to loss of *VdTrx1* (Figures 6A,B). Further, supplementation of exogenous reduced sulfur (Na_2_SO_3_) and cysteine into MM medium could compensate for the defect seen in Δ*VdTrx1* (Figure 6C), suggesting that VdTrx1 is involved in sulfite assimilation in *V. dahliae*.

The effect of *VdTrx1* disruption on conidia production was also studied, revealing a major role of VdTrx1 in conidiation. The Δ*VdTrx1* strain produced 81–85% fewer conidia than the wild-type strain. Although the complemented strain produced more conidia than Δ*VdTrx1*, it did not reach the level of wild-type strain (Figure 6D). Together, these results indicated that VdTrx1 is not involved in cell-wall and hyperosmotic stress tolerance but is critical for sulfite assimilation and conidiation in *V. dahliae*.

### 3.7. VdTrx1 is required for virulence in *Verticillium dahliae*

To examine a possible contribution of VdTrx1 to the virulence of *V. dahliae*, *VdTrx1* transcript levels were quantified by RT-qPCR *in planta* during infection of cotton roots. The results showed that the expression of *VdTrx1* was upregulated after inoculation, reaching the maximum at 4 dpi, and suggesting that *VdTrx1* responds to plant inoculation and may play an important role in pathogenicity (Supplementary Figure S4A). To investigate whether VdTrx1 plays a role during the initial colonization of *V. dahliae*, penetration ability was examined by incubation of all the transformants on cellophane membranes which were overlaid on the minimal medium (MM). Although Δ*VdTrx1* could not form normal aerial hyphae on this medium, they were capable of penetrating the cellophane membrane at 4 dpi (Supplementary Figure S4B), indicating that loss of *VdTrx1* does not affect the penetration ability of *V. dahliae*.

**Figure 7:**
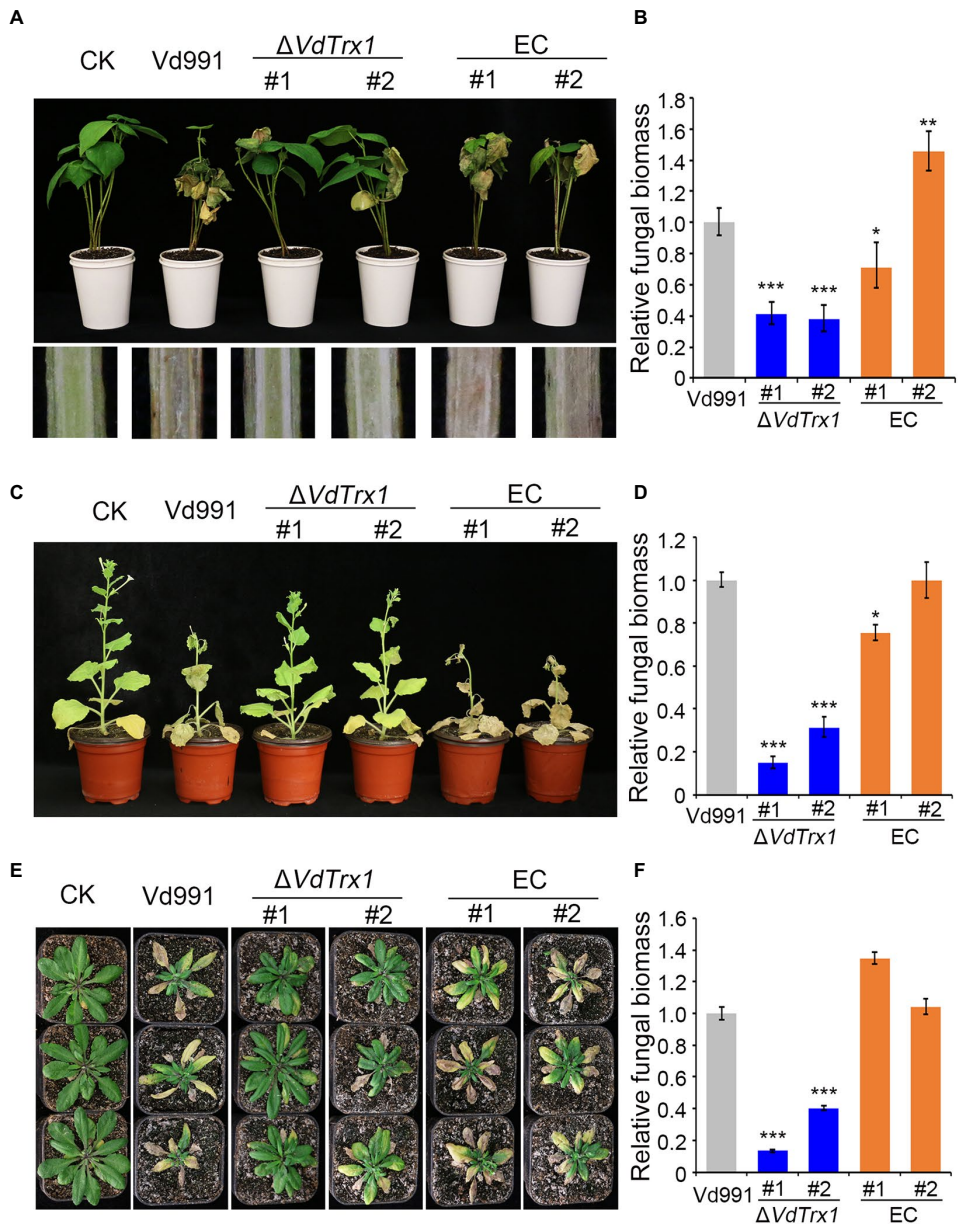
VdTrx1 is required for the full virulence of *V. dahliae*. **(A)** The disease symptoms of cotton seedlings inoculated with sterile water (CK) or the *VdTrx1* deletion strains, complemented strains (EC#1 and #2) and wild-type strain (Vd991) at 21 days post-inoculation (Top). The discoloration of the inoculation shoot longitudinal sections is shown at the bottom. Phenotypes of *N. benthamiana* **(C)** and *A. thaliana* **(E)** plants inoculated with indicated strains. The fungal biomasses of each fungal strain in cotton **(B)**, *N. benthamiana* **(D)** and *A. thaliana* **(F)** plants were determined by qPCR. Error bars represent standard deviations (*n* = 3). Asterisks *** indicate significant differences at (*p* < 0.001) based on the Student’s *t*-test.

Further, the natural cotton host (*Gossypium hirsutum*) and the model plant species (*Arabidopsis thaliana* and *Nicotiana benthamiana*) were used to test the pathogenicity of the Δ*VdTrx1* strain and other *V. dahliae* strains. As expected, cotton inoculated with the wild-type strain showed typical disease symptoms, including wilting of leaves and vascular discoloration. In contrast, in cotton plants that were infected with the Δ*VdTrx1* strain, the disease severity was reduced, accompanied with a significant decrease in fungal biomass *in planta* of 61% compared with the biomass in the plants infected with the wild-type strain (Figure 7A). The virulence and fungal biomass of the complemented strains were comparable to those of the wild-type strain (Figure 7B). Like cotton, targeted deletion of *VdTrx1* resulted in markedly compromised virulence on both *Arabidopsis thaliana* plants and *N. benthamiana*, which were confirmed not only by observations of disease symptoms after plants were inoculated with the various fungal genotypes but also by measurements of fungal biomass *in planta* by quantitative PCR. All pathogenic defects of Δ*VdTrx1* were restored by complementation of *VdTrx1* (Figures 7C–F). Overall, VdTrx1 is required for virulence not only on the natural host of cotton from which the original wild-type isolate used in this study was derived, but also on two other host plant species.

## 4. Discussion

Although thioredoxins lacking a signal peptide have been considered as unconventional secreted proteins in mammalian cells, there has not been similar investigations on the secretory characteristics of thioredoxin in plant pathogenic fungi. In this study, we characterized the unconventionally secreted thioredoxin VdTrx1 and investigated its role in *V. dahliae*-plant interactions using a combination of approaches. The virulence function of VdTrx1 was demonstrated on three different hosts.

Secreted proteins typically carry an *N*-terminal signal peptide in eukaryotic cells and are transported through the conventional ER-Golgi secretion pathway. The majority of identified extracellular proteins in eukaryotes are secreted following this paradigm. However, proteomics analyses have revealed that a high percentage of proteins lacking signal peptides in the secretomes of fungi, including fungal phytopathogens (Rampitsch et al., 2013; González-Fernández et al., 2015). Recently, several studies have revealed the functions of unconventionally secreted proteins involved in plant-microbe interactions. For example, though the *Phytophthora sojae* isochorismatase PsIsc1 lacks a signal peptide, it can be secreted and translocated into soybean cells to suppress salicylate-mediated innate immunity *in planta* by hydrolyzing isochorismate, a precursor of salicylate (Liu et al., 2014). *Magnaporthe oryzae* oxysterol-binding protein-related proteins (MoORPs), normally described as intracellular proteins, are detected in intercellular fluids from barley plants infected with the fungus (Chen et al., 2022). MoORPs act as PAMP molecules to modulate plant innate immunity and promote virulence in *M. oryzae* (Chen et al., 2022). Two independent proteomics analysis also revealed that there are 56 and 99 potential proteins lacking signal peptides in the exoproteome of *V. dahliae* strains Vd07038 and Vd991, respectively, implying that unconventionally secreted proteins also play important roles during host- *V. dahliae* interactions (Chu et al., 2015; Chen et al., 2016). As an analogous analysis of *Phytophthora sojae* PsIsc1, the effector VdIsc1 in *V. dahliae* lacks a signal peptide and also plays a role in inhibiting salicylic acid-mediated plant immune response (Liu et al., 2014). Our previous study also found superoxide dismutase VdSOD1 lacking a signal peptide can be dispatched from *V. dahliae* cells and transferred into host apoplastic region to detoxify host-generated ROS in the form of $O_{2}^{-}$ (Tian et al., 2021b). In this study, the presence of VdTrx1 in the culture filtrate was detected by western blot *in vitro*, and its apoplastic localization *in planta* was also observed by live-cell imaging, providing concrete evidence that VdTrx1 is an unconventionally secreted protein (Figure 3).

The removal of deleterious oxidants is a conserved function of thioredoxin (Koháryová and Kolárová, 2008). Not surprisingly, VdTrx1 functions in scavenging extracellular H_2_O_2_, further reducing the adverse effects of plant-derived ROS on pathogen (Figure 5B). Following the VdSOD1 scenario, the current research provided another example of an unconventionally secreted protein with a ROS-detoxifying function. The advantage of unconventional secretion of antioxidants may be that the oxidative environment in the lumen of the ER may result in the inactivation of sets of enzymes. In addition, cell starvation could lead to conventional protein secretion inhibition, while there is a net increase in the secretion of proteins lacking the signal peptide through an unconventional secretion pathway (Cruz-Garcia et al., 2020). *V. dahliae* colonizes the vascular bundles of plants, which is a nutrition-poor niche (Klosterman et al., 2011). Thus, conventional ER-Golgi secretion systems may be limited during infection and unconventional secretion of pathogens becomes a necessary complementary pathway to dispatch antioxidants for ROS-detoxification in vascular tissue. The findings indicate that searches for extracellular proteins in fungal plant pathogens, which may be pathogenicity factors, should not be restricted to those bearing signal peptides.

At present, we are at the beginning stage of understanding the molecular mechanisms of secretion pathways for cargo proteins lacking signal peptides. The TMED10 of mice cells acts as a protein channel, which directly facilitates the translocation of proteins lacking a signal peptide into the ER-Golgi intermediate compartment (ERGIC), and where the leaderless cargo enters other unknown vesicle carriers to be delivered out of the cell (Zhang et al., 2020). In *S. cerevisiae*, acyl-coenzyme A binding protein Acb1 and SOD1 lacking signal peptides are both captured into a cup-shape like membrane compartment called CUPS, which serves as a sorting station prior to their release into the extracellular space (Cruz-Garcia et al., 2017). Our previous research had shown that the secretion of VdSOD1 of *V. dahliae* requires the participation of VdGRASP, whose orthologous protein plays important roles in the formation and maturation of CUPS in *S. cerevisiae* (Tian et al., 2021b). However, the current study indicated the extracellular release of VdTrx1 is not in a VdGRASP dependent manner, but relies on VdVps36, which is a component of the ESCRT-II complex (Figures 4A,B). Thus, *V. dahliae* likely employs diverse unconventional secretory routes to translocate virulence-related proteins that lack signal peptides out of the cell.

Thioredoxins are ubiquitous small proteins with a redox-active site, which have been conserved throughout evolution in eukaryotes. As hydrogen donors, thioredoxin systems regulate many substrates including peroxiredoxin, an enzyme involved in the reduction of H_2_O_2_; ribonucleotide reductase, required for dNTP synthesis; and PAPS reductase, which contributes to sulfate assimilation (Meyer et al., 2009). In this study, we found that Δ*VdTrx1* showed a reduced radial growth rate accompanied by impaired aerial hyphae in the medium absence of ${\mathrm{SO}}_{3}^{2-}$, indicating that VdTrx1 contributes to sulfite assimilation in *V. dahliae*, consistent with previous results obtained from analyses of other fungal phytopathogens such as *M. oryzae* (Wang et al., 2017) and *Podospora anserina* (Malagnac et al., 2007).

There is growing evidence that thioredoxin directly detoxifies ROS or acts as a regulator of ROS-induced signal transduction pathways. For instance, when the function of *S. sclerotiorum* SsTrx1 is inhibited by gene silencing, higher ROS accumulation was observed after inoculation of *A. thaliana*, and the direct role of *S. sclerotiorum* SsTrx1 in ROS detoxification was demonstrated, providing protection of the pathogen against oxidative stress (Rana et al., 2021). In the human fungal pathogen *C. albicans*, Trx1 regulates three distinct H_2_O_2_ signaling proteins including protein kinases (Hog1 and Rad53) and the transcription factor Cap1 to promote *C. albicans* survival in the host (da Silva Dantas et al., 2010). The thioredoxin protein of *M. oryzae* has two of the above-mentioned functions. On the one hand, MoTRX2 is important for the scavenging of ROS in the plant-fungus interaction (Wang et al., 2017); on the other hand, by directly interacting with kinase MoMst7, MoTRX2 regulates the activation of MAPK pathway in the pathogen that regulates its invasive growth and its ability to infect the plant (Zhang et al., 2016). In *V. dahliae*, members of MAPK pathway (VdHog1, VdPbs2 and VdSsk2) are involved in regulating the hyperosmotic stress response and cell-wall integrity (Tian et al., 2016; Wang Y.L. et al., 2016; Yu et al., 2019). In our current research, we showed that *V. dahliae* VdTrx1 is not essential for the response to cell-wall and hyperosmotic stress (Figure 6A). Instead, VdTrx1 functions in scavenging ROS both endogenously and exogenously (Figures 5A,B), suggesting the role of VdTrx1 in promoting pathogenesis may be dependent on its antioxidant functions rather than regulating MAPK signaling. Whether VdTrx1 is involved in the dNTP synthesis in *V. dahliae* requires further investigation.

Thioredoxins are important virulence factors in many pathogenic fungi, including the human pathogen *C. albicans* (da Silva Dantas et al., 2010) and phytopathogenic fungi, such as *M. oryzae* (Wang et al., 2017) and *S. sclerotiorum* (Rana et al., 2021). In our study, we found that *VdTrx1* was clearly induced during infection, suggesting VdTrx1 may play roles in host-pathogen interactions and in the pathogenicity of *V. dahliae* (Supplementary Figure S4A). Deletion of *VdTrx1* alleviated disease symptoms and reduced fungal biomass in three different hosts, suggesting that VdTrx1 was required for the virulence of *V. dahliae* (Figures 7A–C). Penetration assays showed that loss of *VdTrx1* did not damage the ability of the mutant strain to penetrate cellophane membrane, suggesting Δ*VdTrx1* maintained the ability to infect and initially colonize host tissue (Supplementary Figure S4B). The reduced virulence of *VdTrx1* deletion mutant was likely due to its decreased viability in host vascular tissue (Rouached et al., 2009). First, loss-of-function of *VdTrx1* reduces the pathogen to counter the toxicity of host-derived ROS (Figure 5B). Secondly, ${\mathrm{SO}}_{4}^{2-}$, absorbed by plant root from the soil, is the main form of sulfur in plant vascular tissue. Loss of VdTrx1, which is involved in the reduction process of ${\mathrm{SO}}_{4}^{2-}$, may render the Δ*VdTrx1* strain unable to utilize sulfur effectively for normal growth (Figures 6A,B). Thirdly, according to studies of the invasion progress of *V. dahliae* on host plants, after successful epidermal invasion, conidia are formed in the xylem vessels to promote systemic propagation vertically (Tian and Kong, 2022). The decrease of sporulation ability of Δ*VdTrx1* may slow down this invasive proliferation process (Figure 6D).

**Figure 8:**
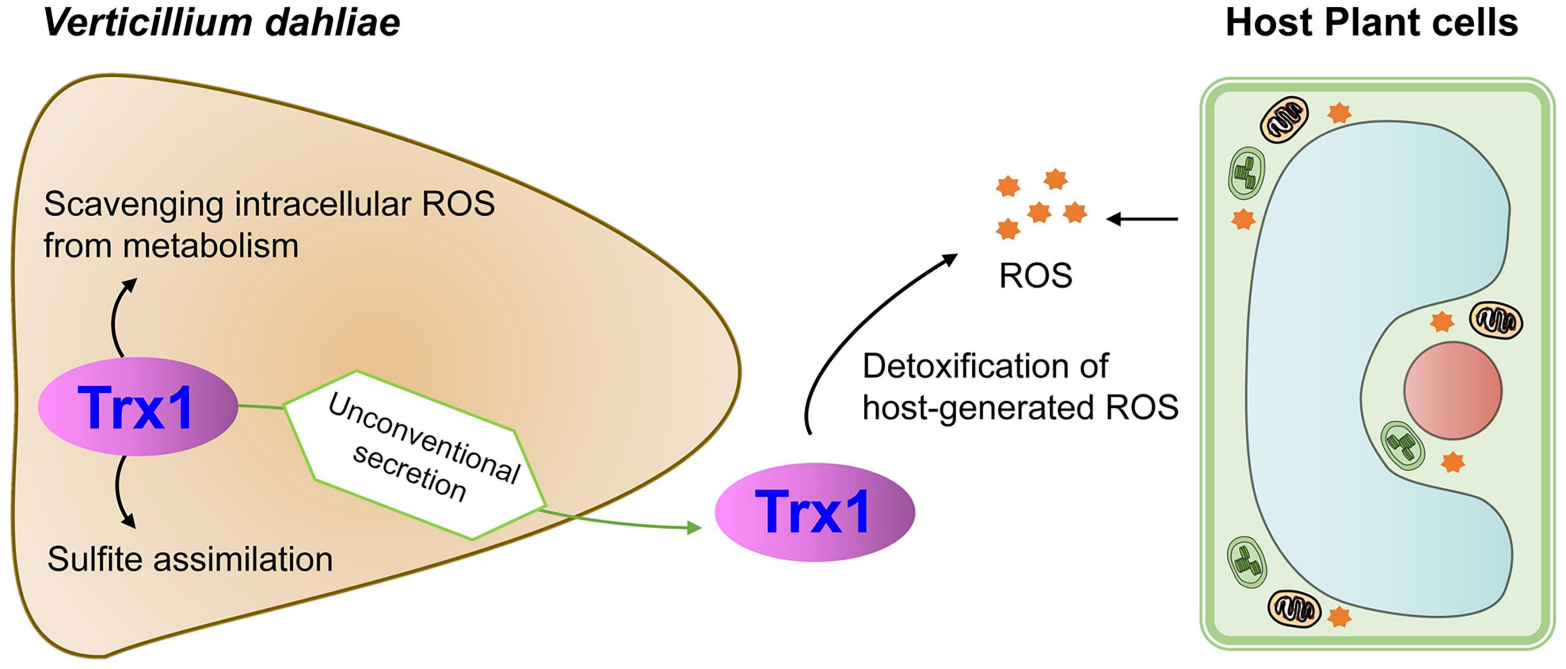
Proposed model of VdTrx1 function during *V. dahliae*-host interactions.

In conclusion, signal-peptide-lacking VdTrx1 is an unconventionally secreted thioredoxin with biological functions both inside and outside of the cells. Its presence in fungal tissue involves in scavenging intracellular ROS and sulfite assimilation. VdTrx1 can also be secreted unconventionally depending on VdVps36, detoxifying host-generated ROS during pathogen-host interactions (Figure 8). VdTrx1 is necessary for full virulence of *V. dahliae* on susceptible hosts.

## Data availability statement

The datasets presented in this study can be found in online repositories. The names of the repository/repositories and accession number(s) can be found in the article/Supplementary material.

## Author contributions

J-YC and LT conceived and designed the experiments. JZ, LT, J-JL, and HZ performed the experiments. D-DZ analyzed the data. LT, D-DZ, and JZ wrote the initial draft. J-YC, KS, SK, and X-FD wrote, reviewed, and edited the manuscript. All authors contributed to the article and approved the submitted version.

## Funding

This research was funded by the National Key Research and Development Program of China (2022YFD1400300, 2022YFE0111300, 2022YFE0130800), the National Natural Science Foundation of China (32270212, 31972228, 31970142), the Agricultural Sciences Talent Program CAAS to J-YC, the Agricultural Science and Technology Innovation Program grant to J-YC, and Shandong Provincial Natural Science Foundation (ZR2020MC115).

## Conflict of interest

The authors declare that the research was conducted in the absence of any commercial or financial relationships that could be construed as a potential conflict of interest.

## Publisher’s note

All claims expressed in this article are solely those of the authors and do not necessarily represent those of their affiliated organizations, or those of the publisher, the editors and the reviewers. Any product that may be evaluated in this article, or claim that may be made by its manufacturer, is not guaranteed or endorsed by the publisher.

## Supplementary material

The Supplementary material for this article can be found online at: https://www.frontiersin.org/articles/10.3389/fmicb.2023.1130468/full#supplementary-material

## Footnotes

^1^ https://www.ncbi.nlm.nih.gov/

^2^ http://www.mbio.ncsu.edu/bioedit/

## References

[ref1] Almagro Armenteros J. J. Tsirigos K. D. Sønderby C. K. Petersen T. N. Winther O. Brunak S.. (2019). SignalP 5.0 improves signal peptide predictions using deep neural networks. Nat. Biotechnol. 37, 420–423. doi: 10.1038/s41587-019-0036-z, PMID: 30778233

[ref2] Bendtsen J. D. Jensen L. J. Blom N. Von Heijne G. Brunak S. (2004). Feature-based prediction of non-classical and leaderless protein secretion. Protein Eng. Des. Sel. 17, 349–356. doi: 10.1093/protein/gzh037, PMID: 15115854

[ref3] Berendt U. Haverkamp T. Prior A. Schwenn J. D. (1995). Reaction mechanism of thioredoxin: 3′-phospho-adenylylsulfate reductase investigated by site-directed mutagenesis. Eur. J. Biochem. 233, 347–356. doi: 10.1111/j.1432-1033.1995.347_1.x, PMID: 7588765

[ref4] Broxton C. N. Culotta V. C. (2016). SOD enzymes and microbial pathogens: surviving the oxidative storm of infection. PLoS Pathog. 12:e1005295. doi: 10.1371/journal.ppat.1005295, PMID: 26742105 PMC4712152

[ref5] Bui T. T. Harting R. Braus-Stromeyer S. A. Tran V. T. Leonard M. Höfer A.. (2019). *Verticillium dahliae* transcription factors Som1 and Vta3 control microsclerotia formation and sequential steps of plant root penetration and colonisation to induce disease. New Phytol. 221, 2138–2159. doi: 10.1111/nph.15514 30290010

[ref6] Camejo D. Guzmán-Cedeño Á. Moreno A. (2016). Reactive oxygen species, essential molecules, during plant-pathogen interactions. Plant Physiol. Biochem. 103, 10–23. doi: 10.1016/j.plaphy.2016.02.035 26950921

[ref7] Chen J. Y. Klosterman S. J. Hu X. P. Dai X. F. Subbarao K. V. (2021). Key insights and research prospects at the dawn of the population genomics era for *Verticillium dahliae*. Annu. Rev. Phytopathol. 59, 31–51. doi: 10.1146/annurev-phyto-020620-121925 33891830

[ref8] Chen J. Y. Liu C. Gui Y. J. Si K. W. Zhang D. D. Wang J.. (2018). Comparative genomics reveals cotton-specific virulence factors in flexible genomic regions in *Verticillium dahliae* and evidence of horizontal gene transfer from *Fusarium*. New Phytol. 217, 756–770. doi: 10.1111/nph.14861, PMID: 29084346 PMC5765495

[ref9] Chen J. Y. Xiao H. L. Gui Y. J. Zhang D. D. Li L. Bao Y. M.. (2016). Characterization of the *Verticillium dahliae* exoproteome involves in pathogenicity from cotton-containing medium. Front. Microbiol. 7:1709. doi: 10.3389/fmicb.2016.01709, PMID: 27840627 PMC5083787

[ref10] Chen M. M. Yang S. R. Wang J. Fang Y. L. Peng Y. L. Fan J. (2022). Fungal oxysterol-binding protein-related proteins promote pathogen virulence and activate plant immunity. J. Exp. Bot. 73, 2125–2141. doi: 10.1093/jxb/erab530 34864987

[ref11] Chu J. Li W. F. Cheng W. Lu M. Zhou K. H. Zhu H. Q.. (2015). Comparative analyses of secreted proteins from the phytopathogenic fungus *Verticillium dahliae* in response to nitrogen starvation. Biochim. Biophys. Acta 1854, 437–448. doi: 10.1016/j.bbapap.2015.02.004 25698221

[ref12] Cruz-Garcia D. Brouwers N. Duran J. M. Mora G. Curwin A. J. Malhotra V. (2017). A diacidic motif determines unconventional secretion of wild-type and ALS-linked mutant SOD1. J. Cell Biol. 216, 2691–2700. doi: 10.1083/jcb.201704056, PMID: 28794127 PMC5584182

[ref13] Cruz-Garcia D. Brouwers N. Malhotra V. Curwin A. J. (2020). Reactive oxygen species triggers unconventional secretion of antioxidants and Acb1. J. Cell Biol. 219:e201905028. doi: 10.1083/jcb.201905028 32328640 PMC7147093

[ref14] da Silva Dantas A. Patterson M. J. Smith D. A. Maccallum D. M. Erwig L. P. Morgan B. A.. (2010). Thioredoxin regulates multiple hydrogen peroxide-induced signaling pathways in *Candida albicans*. Mol. Cell. Biol. 30, 4550–4563. doi: 10.1128/mcb.00313-10, PMID: 20679492 PMC2950526

[ref15] Ferro-Novick S. Brose N. (2013). Nobel 2013 physiology or medicine: traffic control system within cells. Nature 504:98. doi: 10.1038/504098a 24305158

[ref16] Gayoso C. Pomar F. Novo-Uzal E. Merino F. de Ilárduya O. M. (2010). The *Ve*-mediated resistance response of the tomato to *Verticillium dahliae* involves H_2_O_2_, peroxidase and lignins and drives *PAL* gene expression. BMC Plant Biol. 10:232. doi: 10.1186/1471-2229-10-232 20977727 PMC3095318

[ref17] González-Fernández R. Valero-Galván J. Gómez-Gálvez F. J. Jorrín-Novo J. V. (2015). Unraveling the in vitro secretome of the phytopathogen *Botrytis cinerea* to understand the interaction with its hosts. Front. Plant Sci. 6:839. doi: 10.3389/fpls.2015.00839, PMID: 26500673 PMC4598570

[ref18] Gui Y. J. Chen J. Y. Zhang D. D. Li N. Y. Li T. G. Zhang W. Q.. (2017). *Verticillium dahliae* manipulates plant immunity by glycoside hydrolase 12 proteins in conjunction with carbohydrate-binding module 1. Environ. Microbiol. 19, 1914–1932. doi: 10.1111/1462-2920.13695, PMID: 28205292

[ref19] Heller J. Tudzynski P. (2011). Reactive oxygen species in phytopathogenic fungi: signaling, development, and disease. Annu. Rev. Phytopathol. 49, 369–390. doi: 10.1146/annurev-phyto-072910-095355 21568704

[ref20] Holmgren A. (1989). Thioredoxin and glutaredoxin systems. J. Biol. Chem. 264, 13963–13966. doi: 10.1016/S0021-9258(18)71625-6 2668278

[ref21] Imlay J. A. (2003). Pathways of oxidative damage. Annu. Rev. Microbiol. 57, 395–418. doi: 10.1146/annurev.micro.57.030502.090938 14527285

[ref22] Jacobs K. A. Collins-Racie L. A. Colbert M. Duckett M. Golden-Fleet M. Kelleher K.. (1997). A genetic selection for isolating cDNAs encoding secreted proteins. Gene 198, 289–296. doi: 10.1016/s0378-1119(97)00330-2 9370294

[ref23] Khang C. H. Park S. Y. Lee Y. H. Kang S. (2005). A dual selection based, targeted gene replacement tool for *Magnaporthe grisea* and *Fusarium oxysporum*. Fungal Genet. Biol. 42, 483–492. doi: 10.1016/j.fgb.2005.03.004, PMID: 15893252

[ref24] Klosterman S. J. Atallah Z. K. Vallad G. E. Subbarao K. V. (2009). Diversity, pathogenicity, and management of verticillium species. Annu. Rev. Phytopathol. 47, 39–62. doi: 10.1146/annurev-phyto-080508-081748 19385730

[ref25] Klosterman S. J. Subbarao K. V. Kang S. Veronese P. Gold S. E. Thomma B. P.. (2011). Comparative genomics yields insights into niche adaptation of plant vascular wilt pathogens. PLoS Pathog. 7:e1002137. doi: 10.1371/journal.ppat.1002137, PMID: 21829347 PMC3145793

[ref26] Koháryová M. Kolárová M. (2008). Oxidative stress and thioredoxin system. Gen. Physiol. Biophys. 27, 71–84. 18645221

[ref27] Lamb C. Dixon R. A. (1997). The oxidative burst in plant disease resistance. Annu. Rev. Plant Physiol. Plant Mol. Biol. 48, 251–275. doi: 10.1146/annurev.arplant.48.1.251 15012264

[ref28] Léveillard T. Aït-Ali N. (2017). Cell signaling with extracellular thioredoxin and thioredoxin-like proteins: insight into their mechanisms of action. Oxidative Med. Cell. Longev. 2017:8475125. doi: 10.1155/2017/8475125, PMID: 29138681 PMC5613632

[ref29] Liu T. Song T. Zhang X. Yuan H. Su L. Li W.. (2014). Unconventionally secreted effectors of two filamentous pathogens target plant salicylate biosynthesis. Nat. Commun. 5:4686. doi: 10.1038/ncomms5686, PMID: 25156390 PMC4348438

[ref30] Livak K. J. Schmittgen T. D. (2001). Analysis of relative gene expression data using real-time quantitative PCR and the 2^−ΔΔCT^ method. Methods 25, 402–408. doi: 10.1006/meth.2001.1262 11846609

[ref31] Lu J. Holmgren A. (2014). The thioredoxin antioxidant system. Free Radic. Biol. Med. 66, 75–87. doi: 10.1016/j.freeradbiomed.2013.07.036 23899494

[ref32] Malagnac F. Klapholz B. Silar P. (2007). PaTrx1 and PaTrx3, two cytosolic thioredoxins of the filamentous ascomycete *Podospora anserina* involved in sexual development and cell degeneration. Eukaryot. Cell 6, 2323–2331. doi: 10.1128/ec.00083-07 17933907 PMC2168258

[ref33] Meyer Y. Buchanan B. B. Vignols F. Reichheld J. P. (2009). Thioredoxins and glutaredoxins: unifying elements in redox biology. Annu. Rev. Genet. 43, 335–367. doi: 10.1146/annurev-genet-102108-134201, PMID: 19691428

[ref34] Palade G. (1975). Intracellular aspects of the process of protein synthesis. Science 189:867. doi: 10.1126/science.189.4206.867-b 17812524

[ref35] Rampitsch C. Day J. Subramaniam R. Walkowiak S. (2013). Comparative secretome analysis of *Fusarium graminearum* and two of its non-pathogenic mutants upon deoxynivalenol induction in vitro. Proteomics 13, 1913–1921. doi: 10.1002/pmic.201200446, PMID: 23512867

[ref36] Rana K. Ding Y. Banga S. S. Liao H. Zhao S. Yu Y.. (2021). *Sclerotinia sclerotiorum* Thioredoxin1 (SsTrx1) is required for pathogenicity and oxidative stress tolerance. Mol. Plant Pathol. 22, 1413–1426. doi: 10.1111/mpp.13127 34459563 PMC8518572

[ref37] Rolke Y. Liu S. Quidde T. Williamson B. Schouten A. Weltring K. M.. (2004). Functional analysis of H_2_O_2_-generating systems in *Botrytis cinerea*: the major cu-Zn-superoxide dismutase (BCSOD1) contributes to virulence on French bean, whereas a glucose oxidase (BCGOD1) is dispensable. Mol. Plant Pathol. 5, 17–27. doi: 10.1111/j.1364-3703.2004.00201.x, PMID: 20565578

[ref38] Rouached H. Secco D. Arpat A. B. (2009). Getting the most sulfate from soil: regulation of sulfate uptake transporters in *Arabidopsis*. J. Plant Physiol. 166, 893–902. doi: 10.1016/j.jplph.2009.02.016 19375816

[ref39] Segal L. M. Wilson R. A. (2018). Reactive oxygen species metabolism and plant-fungal interactions. Fungal Genet. Biol. 110, 1–9. doi: 10.1016/j.fgb.2017.12.003, PMID: 29225185

[ref40] Staerck C. Gastebois A. Vandeputte P. Calenda A. Larcher G. Gillmann L.. (2017). Microbial antioxidant defense enzymes. Microb. Pathog. 110, 56–65. doi: 10.1016/j.micpath.2017.06.015 28629723

[ref41] Sun M. Zhang Z. Ren Z. Wang X. Sun W. Feng H.. (2021). The GhSWEET42 glucose transporter participates in *Verticillium dahliae* infection in cotton. Front. Plant Sci. 12:690754. doi: 10.3389/fpls.2021.690754, PMID: 34386026 PMC8353158

[ref42] Tian L. Huang C. M. Zhang D. D. Li R. Chen J. Y. Sun W. X.. (2021a). Extracellular superoxide dismutase VdSOD5 is required for virulence in *Verticillium dahliae*. J. Integr. Agric. 20, 1858–1870. doi: 10.1016/S2095-3119(20)63353-6

[ref43] Tian J. Kong Z. (2022). Live-cell imaging elaborating epidermal invasion and vascular proliferation/colonization strategy of *Verticillium dahliae* in host plants. Mol. Plant Pathol. 23, 895–900. doi: 10.1111/mpp.13212 35322912 PMC9104255

[ref44] Tian L. Li J. Huang C. Zhang D. Xu Y. Yang X.. (2021b). Cu/Zn superoxide dismutase (VdSOD1) mediates reactive oxygen species detoxification and modulates virulence in *Verticillium dahliae*. Mol. Plant Pathol. 22, 1092–1108. doi: 10.1111/mpp.13099, PMID: 34245085 PMC8359004

[ref45] Tian L. Y. Wang Y. L. Yu J. Xiong D. G. Zhao H. J. Tian C. M. (2016). The mitogen-activated protein kinase kinase VdPbs2 of *Verticillium dahliae* regulates microsclerotia formation, stress response, and plant infection. Front. Microbiol. 7:1532. doi: 10.3389/fmicb.2016.01532, PMID: 27729908 PMC5037172

[ref46] Tran V. T. Braus-Stromeyer S. A. Kusch H. Reusche M. Kaever A. Kühn A.. (2014). *Verticillium* transcription activator of adhesion Vta2 suppresses microsclerotia formation and is required for systemic infection of plant roots. New Phytol. 202, 565–581. doi: 10.1111/nph.12671, PMID: 24433459

[ref47] Verdoucq L. Vignols F. Jacquot J. P. Chartier Y. Meyer Y. (1999). *In vivo* characterization of a thioredoxin h target protein defines a new peroxiredoxin family. J. Biol. Chem. 274, 19714–19722. doi: 10.1074/jbc.274.28.19714 10391912

[ref48] Vignols F. Bréhélin C. Surdin-Kerjan Y. Thomas D. Meyer Y. (2005). A yeast two-hybrid knockout strain to explore thioredoxin-interacting proteins *in vivo*. Proc. Natl. Acad. Sci. U. S. A. 102, 16729–16734. doi: 10.1073/pnas.0506880102, PMID: 16272220 PMC1283818

[ref49] Wakasugi N. Tagaya Y. Wakasugi H. Mitsui A. Maeda M. Yodoi J.. (1990). Adult T-cell leukemia-derived factor/thioredoxin, produced by both human T-lymphotropic virus type I- and Epstein-Barr virus-transformed lymphocytes, acts as an autocrine growth factor and synergizes with interleukin 1 and interleukin 2. Proc. Natl. Acad. Sci. U. S. A. 87, 8282–8286. doi: 10.1073/pnas.87.21.8282, PMID: 2172979 PMC54939

[ref50] Wang Y. L. Deng C. L. Tian L. Y. Xiong D. G. Tian C. M. Klosterman S. J. (2018). The transcription factor VdHapX controls iron homeostasis and is crucial for virulence in the vascular pathogen *Verticillium dahliae*. mSphere 3:e00400-18. doi: 10.1128/mSphere.00400-18 30185514 PMC6126142

[ref51] Wang Y. L. Tian L. Y. Xiong D. G. Klosterman S. J. Xiao S. X. Tian C. M. (2016). The mitogen-activated protein kinase gene, *VdHog1*, regulates osmotic stress response, microsclerotia formation and virulence in *Verticillium dahliae*. Fungal Genet. Biol. 88, 13–23. doi: 10.1016/j.fgb.2016.01.011, PMID: 26812120

[ref52] Wang J. Tian L. Zhang D. D. Short D. P. G. Zhou L. Song S. S.. (2018). SNARE-encoding genes *VdSec22* and *VdSso1* mediate protein secretion required for full virulence in *Verticillium dahliae*. Mol. Plant-Microbe Interact. 31, 651–664. doi: 10.1094/mpmi-12-17-0289-r 29419372

[ref53] Wang D. Tian L. Zhang D. D. Song J. Song S. S. Yin C. M.. (2020). Functional analyses of small secreted cysteine-rich proteins identified candidate effectors in *Verticillium dahliae*. Mol. Plant Pathol. 21, 667–685. doi: 10.1111/mpp.12921, PMID: 32314529 PMC7170778

[ref54] Wang S. Wu X. M. Liu C. H. Shang J. Y. Gao F. Guo H. S. (2020). *Verticillium dahliae* chromatin remodeling facilitates the DNA damage repair in response to plant ROS stress. PLoS Pathog. 16:e1008481. doi: 10.1371/journal.ppat.1008481, PMID: 32298394 PMC7188298

[ref55] Wang Y. M. Wu J. N. Park Z. Y. Kim S. G. Rakwal R. Agrawal G. K.. (2011). Comparative secretome investigation of *Magnaporthe oryzae* proteins responsive to nitrogen starvation. J. Proteome Res. 10, 3136–3148. doi: 10.1021/pr200202m 21563842

[ref56] Wang S. Xing H. Y. Hua C. L. Guo H. S. Zhang J. (2016). An improved single-step cloning strategy simplifies the *Agrobacterium tumefaciens*-mediated transformation (ATMT)-based gene-disruption method for *Verticillium dahliae*. Phytopathology 106, 645–652. doi: 10.1094/phyto-10-15-0280-r, PMID: 26780432

[ref57] Wang B. N. Yang X. F. Zeng H. M. Liu H. Zhou T. T. Tan B. B.. (2012). The purification and characterization of a novel hypersensitive-like response-inducing elicitor from *Verticillium dahliae* that induces resistance responses in tobacco. Appl. Microbiol. Biotechnol. 93, 191–201. doi: 10.1007/s00253-011-3405-1 21691787

[ref58] Wang J. Z. Yin Z. Y. Tang W. Cai X. J. Gao C. Y. Zhang H. F.. (2017). The thioredoxin MoTrx2 protein mediates reactive oxygen species (ROS) balance and controls pathogenicity as a target of the transcription factor MoAP1 in *Magnaporthe oryzae*. Mol. Plant Pathol. 18, 1199–1209. doi: 10.1111/mpp.12484, PMID: 27560036 PMC6638232

[ref59] Yao S. H. Guo Y. Wang Y. Z. Zhang D. Xu L. Tang W. H. (2016). A cytoplasmic cu-Zn superoxide dismutase SOD1 contributes to hyphal growth and virulence of *Fusarium graminearum*. Fungal Genet. Biol. 91, 32–42. doi: 10.1016/j.fgb.2016.03.006, PMID: 27037138

[ref60] Yu J. Li T. Y. Tian L. Tang C. Klosterman S. J. Tian C. M.. (2019). Two *Verticillium dahliae* MAPKKKs, VdSsk2 and VdSte11, have distinct roles in pathogenicity, microsclerotial formation, and stress adaptation. mSphere 4:e00426-19. doi: 10.1128/mSphere.00426-19 31292234 PMC6620378

[ref61] Zhang S. J. Jiang C. Zhang Q. Qi L. L. Li C. H. Xu J. R. (2016). Thioredoxins are involved in the activation of the *PMK1* MAP kinase pathway during appressorium penetration and invasive growth in *Magnaporthe oryzae*. Environ. Microbiol. 18, 3768–3784. doi: 10.1111/1462-2920.13315 27059015

[ref62] Zhang M. Liu L. Lin X. Wang Y. Li Y. Guo Q.. (2020). A translocation pathway for vesicle-mediated unconventional protein secretion. Cells 181, 637–652.e15. doi: 10.1016/j.cell.2020.03.031, PMID: 32272059

[ref63] Zhang Y. Wang X. F. Rong W. Yang J. Li Z. K. Wu L. Q.. (2017). Histochemical analyses reveal that stronger intrinsic defenses in *Gossypium barbadense* than in *G. hirsutum* are associated with resistance to *Verticillium dahliae*. Mol. Plant-Microbe Interact. 30, 984–996. doi: 10.1094/mpmi-03-17-0067-r, PMID: 28850286

[ref64] Zhang D. D. Wang J. Wang D. Kong Z. Q. Zhou L. Zhang G. Y.. (2019). Population genomics demystifies the defoliation phenotype in the plant pathogen *Verticillium dahliae*. New Phytol. 222, 1012–1029. doi: 10.1111/nph.15672, PMID: 30609067 PMC6594092

[ref65] Zhao L. Poschmann G. Waldera-Lupa D. Rafiee N. Kollmann M. Stühler K. (2019). OutCyte: a novel tool for predicting unconventional protein secretion. Sci. Rep. 9:19448. doi: 10.1038/s41598-019-55351-z, PMID: 31857603 PMC6923414

[ref66] Zhou L. Zhao J. Guo W. Zhang T. (2013). Functional analysis of autophagy genes via agrobacterium-mediated transformation in the vascular wilt fungus Verticillium dahliae. J. Genet. Genomics 40, 421–431. doi: 10.1016/j.jgg.2013.04.006, PMID: 23969251

