# Supplementary material for: Thioredoxin VdTrx1, an unconventional secreted protein, is a virulence factor in Verticillium dahliae
Source: Front Microbiol. 2023 Mar 31;14:1130468. doi: 10.3389/fmicb.2023.1130468 (PMC10102666; doi:10.3389/fmicb.2023.1130468)
Supplement: Supplementary file 1 [file Data_Sheet_1.DOCX]

Supplementary Material

Thioredoxin VdTrx1, an unconventional secreted protein, is a virulence factor in *Verticillium dahliae*

**Li Tian†, Jing Zhuang†, Krishna V. Subbarao* and Dan-Dan Zhang***

*** Correspondence:** Dan-Dan Zhang: zhangdandan@caas.cn;

Krishna V. Subbarao : kvsubbarao@ucdavis.edu;

## Supplementary Figures


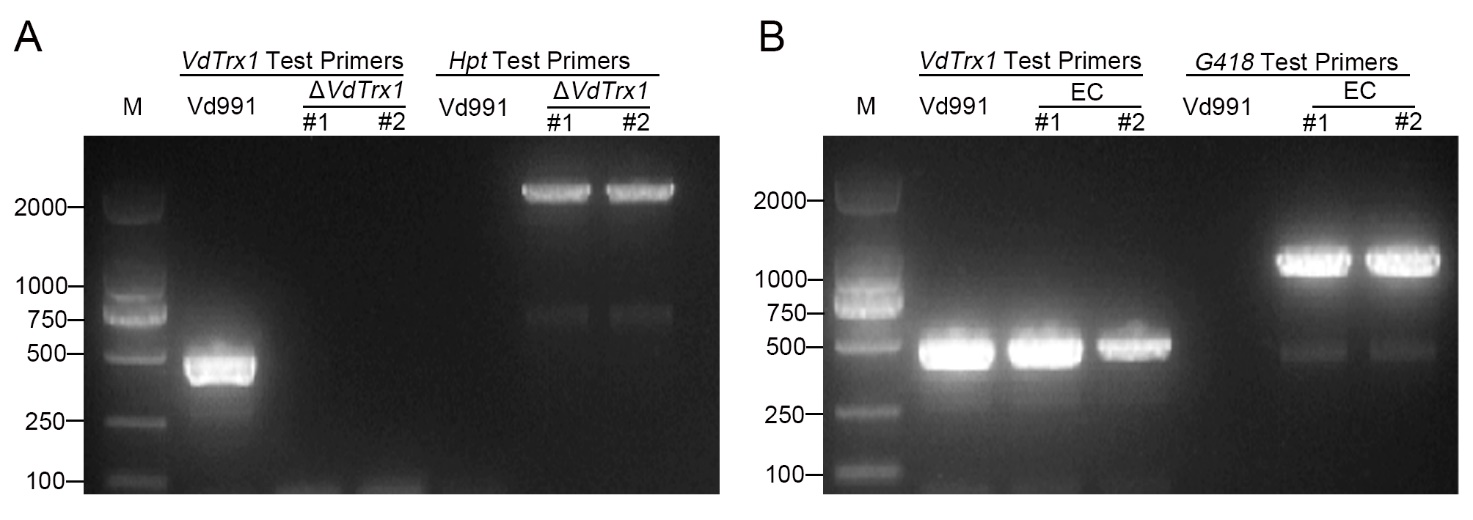


**Figure S1 | Confirmation of *VdTrx1* targeted deletion mutants and *VdTrx1* complementated strains of *V. dahliae*.**

**(A)** Detection of the targeted *VdTrx1* deletion strains by amplifying the coding region of *VdTrx1* and the *Hpt* fragment, respectively. **(B)** Detection of the complemented strains by amplifying a *VdTrx1* internal fragment and the *G418* fragment, respectively.


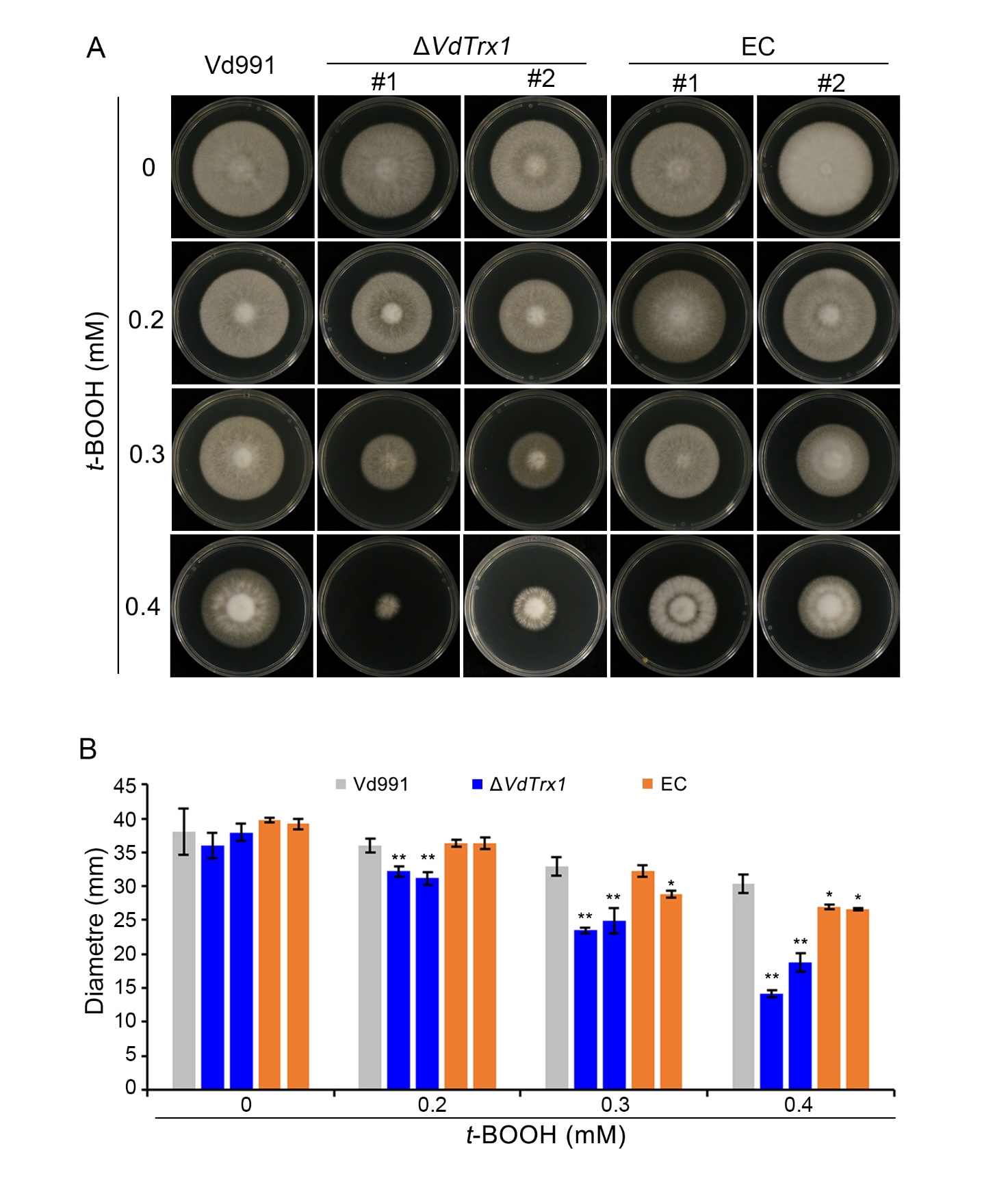


Figure S2 | **The *V. dahliae* VdTrx1 is involved in the response to oxidative stress in media supplemented with tert-Butyl hydroperoxide (t-BOOH).** (**A**) Radial growth of *VdTrx1* deletion strains, complemented strains, and wild-type strain Vd991 on CM medium supplemented with t-BOOH at specified concentrations for 9 days. **(B)** Colony diameters of various *V. dahliae* strains on CM plates containing different concentration of H_2_O_2_ following 9 days incubation. The mean with standard deviation from three biological replicates is shown. Asterisks ** denote significant differences at *P* < 0.01, based on the Student’s *t*-test.


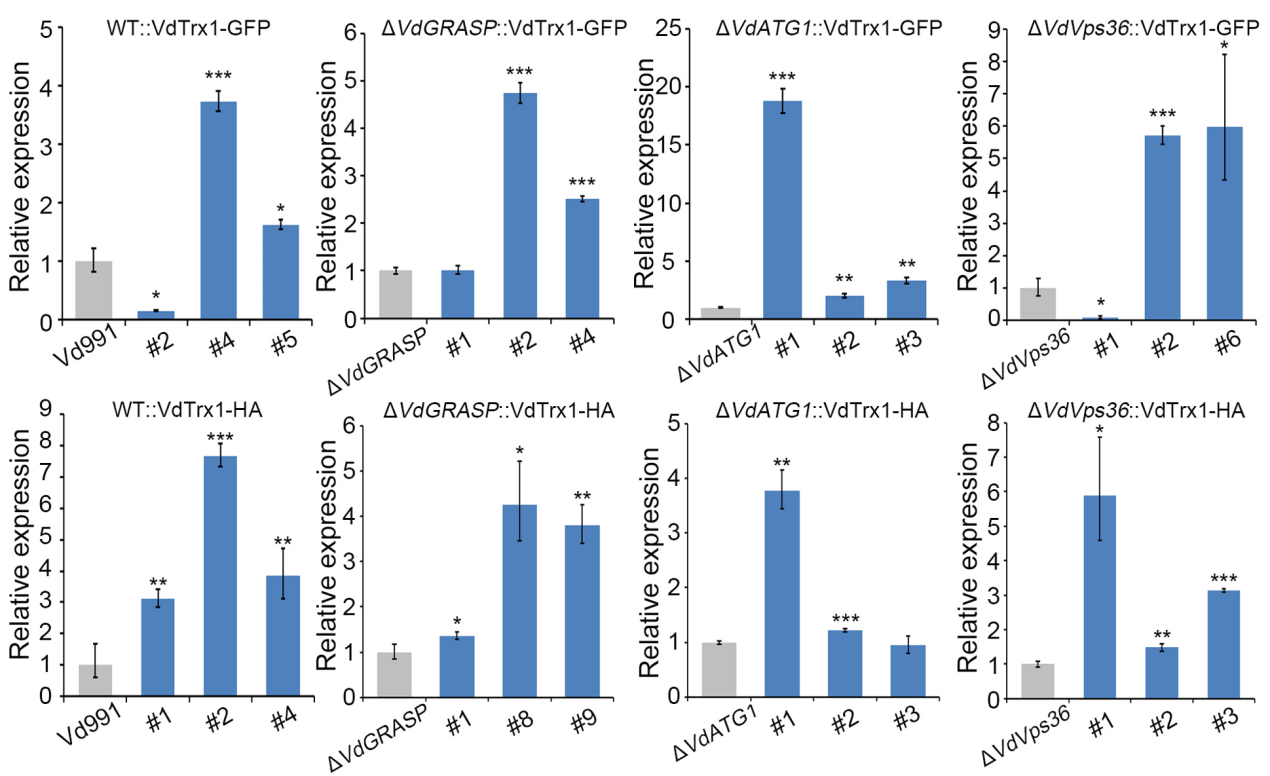


**Figure S3**. **Expression levels of *VdTrx1* in overexpression transformants of *V. dahliae*.** Expression levels of *VdTrx1* of *V. dahliae* in the genetic background shown (as indicated in figure) were determined via reverse transcription-quantitative PCR. The housekeeping gene elongation factor 1-α (*EF-1α*) was used as an endogenous control. Error bars represent the standard deviation of three replicate experiments, and a double asterisk indicates statistical significance (*P*<0.01) based on the Student’s *t*-test. In each *V. dahliae* strain of a different genotype, transformants with the highest expression levels of *VdTrx1* were selected for follow-up studies.


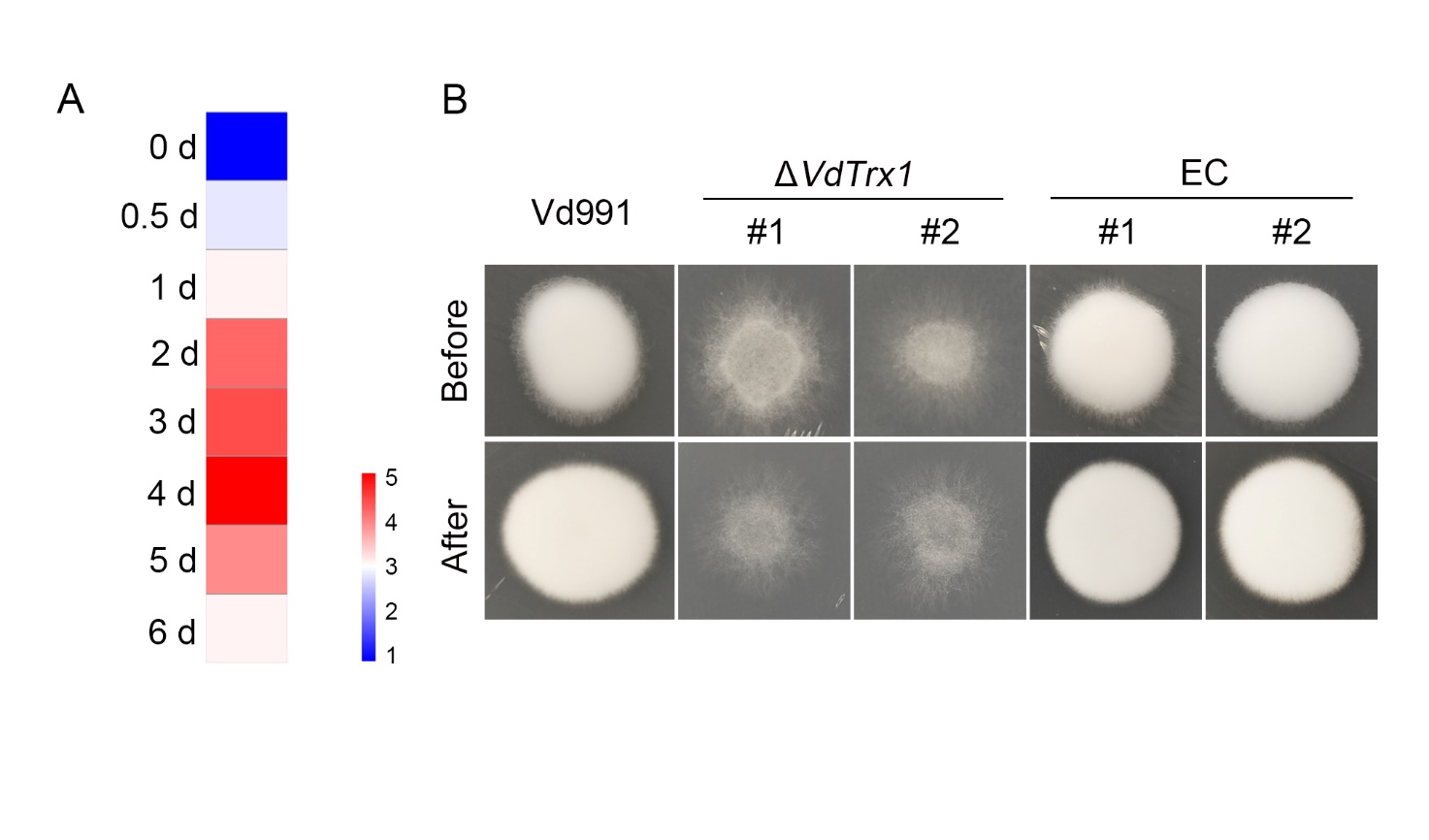


**Figure S4 | Analyses of VdTrx1 expression during infection and its role in penetration**

(A) Reverse transcription-quantitative PCR analyses of the transcript levels of *VdTrx1* during infection of cotton. The expression levels of *VdTrx1* were calculated by comparing the expression levels of *VdTrx1* in infected cotton to the time point of 0 (0 d). and *EF-1α* of *V. dahliae* as an endogenous control. (B) Penetration assay of the wild-type strain Vd991, *VdTrx1* deletion strains and the complemented strains. The strains were all grown on the top of cellophane membranes overlaid onto MM for 4 days at 25°C (“Before” status). The cellophane membranes were then removed from the plates and allowed to incubate for an additional 4 days to determine the cellophane membrane penetration by the presence or absence of hyphal growth on medium (“After” status).

**Table S1 Primers used in this study.**

| **Primer name** | **Primer sequence (5′-3′)** |
| --- | --- |
| *VdTrx1 gene cloning* |  |
| VdTrx1-F | ATGGGCGTCCACAACATCAA |
| VdTrx1-R | TTAGGCGGAGTACTTCAGGA |
| *Cloning VdTrx1 signal peptide* |  |
| SP-VdTrx1-F | GGAATTTTAATTAAGAATTCATGGGCGTCCACAACATCAA |
| SP-VdTrx1-R | CTATAGGGAGAACCTCGAGCGGCGGCGAGGGTGACCTCC |
| VdTrx1-UP-F | GGCACTCGTCCTTCATCCAA |
| *VdTrx1 deletion* |  |
| VdTrx1-UP-R | GCCCAAAAATGCTCCTTCAACGAGTTGATGTTGTGGACGC |
| VdTrx1-Down-F | CCCTGGGTTCGCAAAGATAAGAGGAGATTGTCGGTGCCAA |
| VdTrx1-Down-R | TACTACAGCCACCAGGACGA |
| VdTrx1-Nest-F | GAGGTCGACGGTATCGATAAGCTTTGATATTGAGGCGCGTTCGT |
| VdTrx1-Nest-R | CCAATTTGAGTACCCAATTCGAATTCCCAGGCTGACGAGCTGAG |
| HYG-F | TTGAAGGAGCATTTTTGGGC |
| HYG-R | TTATCTTTGCGAACCCAGGG |
| VdTrx1-Test-F | CCCTGCCATCACATCTAGGC |
| VdTrx1-Test-R | CATTCCATGAAGCGCCATCC |
| *VdTrx1 complementation* |  |
| VdTrx1-C-F | AGCTCGGTACCCGGGGATCCTCTAGAGGCACTCGTCCTTCATCCAA |
| VdTrx1-C-R | TTGCATGCCTGCAGGTCGACTCTAGACCAGGATCTCTTGCGTGGTT |
| *VdTrx1-HA overexpression* |  |
| VdTrx1-HA-F | TACCCAAGCATCGATGAGCTCATGGGCGTCCACAACATCA |
| VdTrx1-HA-R | AACGTTAAGTGGATCTCTAGATTAGGCGTAGTCAGGCACGTCGTAAGGATAGGCGGAGTACTTCAGGAG |
| *VdTrx1-GFP overexpression* |  |
| VdTrx1-GFP-F | ACCCAAGCATCGATGGTACCATGGGCGTCCACAACATCA |
| VdTrx1-GFP-R | CCCTTGCTCACCATGGTACCGGCGGAAGACTTCAGGAG |
| Geneticin-F | ATGATTGAACAAGATGGATTG |
| Geneticin-R | TCAGAAGAACTCGTCAAGAAGG |
| *transcription levels of VdTrx1* |  |
| qVdTrx1-F | CATCCTCGACTGCTTCGCTA |
| qVdTrx1-R | GCCGTCCTTGAAGACCATGA |
| *Fungal biomass detection* |  |
| VdEF-1α-F | TGAGTTCGAGGCTGGTATCT |
| VdEF-1α-R | CACTTGGTGGTGTCCATCTT |
| 18S-F | CGGCTACCACATCCAAGGAA |
| 18S-R | TGTCACTACCTCCCCGTGTCA |
| NbEF-1α-F | TGAGTTCGAGGCTGGTATCT |
| NbEF-1α-R | CACTTGGTGGTGTCCATCTT |
| AtUBQ1-F | TTCCTTGATGATGCTTGCTC |
| AtUBQ1-R | TTGACAGCTCTTGGGTGAAG |

**Table S2 Strains used in this study.**

| **Strain** | **Genotype description** | **Reference** |
| --- | --- | --- |
| ***Verticillium dahliae* strains** |  |  |
| Vd991 | Wild type, highly virulent strain from cotton in China | (Chen et al., 2018) |
| ∆***VdTrx1*** | ***VdTrx1*** deletion mutant | This study |
| EC-1/2 | ***VdTrx1*** complementary strain | This study |
| Δ*VdGRASP* | *VdGRASP* deletion mutant | Unpublished data |
| Δ*VdATG1* | *VdATG1* deletion mutant | Unpublished data |
| Δ*VdVps36* | *VdVps36* deletion mutant | Unpublished data |
| WT::**VdTrx1**-HA | Transformant of Vd991 overexpressing **VdTrx1**-HA fusion protein | This study |
| WT:: **VdTrx1**-GFP | Transformant of Vd991 overexpressing **VdTrx1**-GFP fusion protein | This study |
| Δ*VdGRASP*::**VdTrx1**-HA | Transformant of Δ*VdGRASP* overexpressing **VdTrx1-**HA fusion protein | This study |
| Δ*VdGRASP*::**VdTrx1**-GFP | Transformant of Δ*VdGRASP* overexpressing **VdTrx1**-GFP fusion protein | This study |
| Δ*VdATG1*::**VdTrx1**-HA | Transformant of Δ*VdATG1* overexpressing **VdTrx1-**HA fusion protein | This study |
| Δ*VdATG1*::**VdTrx1**-GFP | Transformant of Δ*VdATG1* overexpressing **VdTrx1**-GFP fusion protein | This study |
| Δ*VdVps36*::**VdTrx1**-HA | Transformant of Δ*VdVps36* overexpressing **VdTrx1-**HA fusion protein | This study |
| Δ*VdVps36*::**VdTrx1**-GFP | Transformant of Δ*VdVps36* overexpressing **VdTrx1**-GFP fusion protein | This study |
| WT::GFP | Transformant of Vd991 overexpressing free GFP protein | (Tian et al., 2021b) |
